# Membrane trafficking alterations in breast cancer progression

**DOI:** 10.3389/fcell.2024.1350097

**Published:** 2024-03-12

**Authors:** Andreia Ferreira, Pedro Castanheira, Cristina Escrevente, Duarte C. Barral, Teresa Barona

**Affiliations:** iNOVA4Health, Faculdade de Ciências Médicas, NMS, FCM, NOVA Medical School, Universidade NOVA de Lisboa, Lisboa, Portugal

**Keywords:** breast cancer, ARF, ARL, RAB, membrane trafficking, invasion, metastasis

## Abstract

Breast cancer (BC) is the most common type of cancer in women, and remains one of the major causes of death in women worldwide. It is now well established that alterations in membrane trafficking are implicated in BC progression. Indeed, membrane trafficking pathways regulate BC cell proliferation, migration, invasion, and metastasis. The 22 members of the ADP-ribosylation factor (ARF) and the >60 members of the rat sarcoma (RAS)-related in brain (RAB) families of small GTP-binding proteins (GTPases), which belong to the RAS superfamily, are master regulators of membrane trafficking pathways. ARF-like (ARL) subfamily members are involved in various processes, including vesicle budding and cargo selection. Moreover, ARFs regulate cytoskeleton organization and signal transduction. RABs are key regulators of all steps of membrane trafficking. Interestingly, the activity and/or expression of some of these proteins is found dysregulated in BC. Here, we review how the processes regulated by ARFs and RABs are subverted in BC, including secretion/exocytosis, endocytosis/recycling, autophagy/lysosome trafficking, cytoskeleton dynamics, integrin-mediated signaling, among others. Thus, we provide a comprehensive overview of the roles played by ARF and RAB family members, as well as their regulators in BC progression, aiming to lay the foundation for future research in this field. This research should focus on further dissecting the molecular mechanisms regulated by ARFs and RABs that are subverted in BC, and exploring their use as therapeutic targets or prognostic markers.

## 1 Introduction

Breast cancer (BC) is the most frequent type of cancer diagnosed in women worldwide. According to Cancer statistics 2023, 31% of newly-diagnosed cancers in women are BC, with a 15% mortality rate (Siegel et al., 2023). Therefore, the mortality and morbidity caused by BC remain high in the female population. BC is a heterogeneous disease that can be classified in different types and subtypes, according to the histological characteristics, behavior and responses to treatment. In general, BC can be divided in non-invasive and invasive carcinomas. Non-invasive BCs can be either ductal carcinomas *in situ* (DCIS) or lobular carcinomas *in situ* (LCIS) (Makki, 2015). Both of these non-invasive forms of BC have the potential to progress to an invasive state, becoming invasive ductal carcinomas (IDCs) or invasive lobular carcinomas (ILs), respectively (Makki, 2015). Molecular subtyping, which is based on the expression of hormone receptors, is another important type of BC classification. The molecular subtypes include: luminal A tumors, which are estrogen receptor (ER)-positive, progesterone receptor (PR)-positive and human epidermal growth factor receptor 2 (HER2)-negative, and have the best prognosis; luminal B tumors, which are ER-positive, have low PR expression, and can be either HER2-positive or HER2-negative; HER2-enriched tumors, which have an amplification of the HER2 gene, do not express ER or PR and are highly proliferative; and triple-negative BC (TNBC), which is defined by the absence of ER, PR and HER2, and has the worst prognosis of all subtypes (Makki, 2015).

**FIGURE 1:**
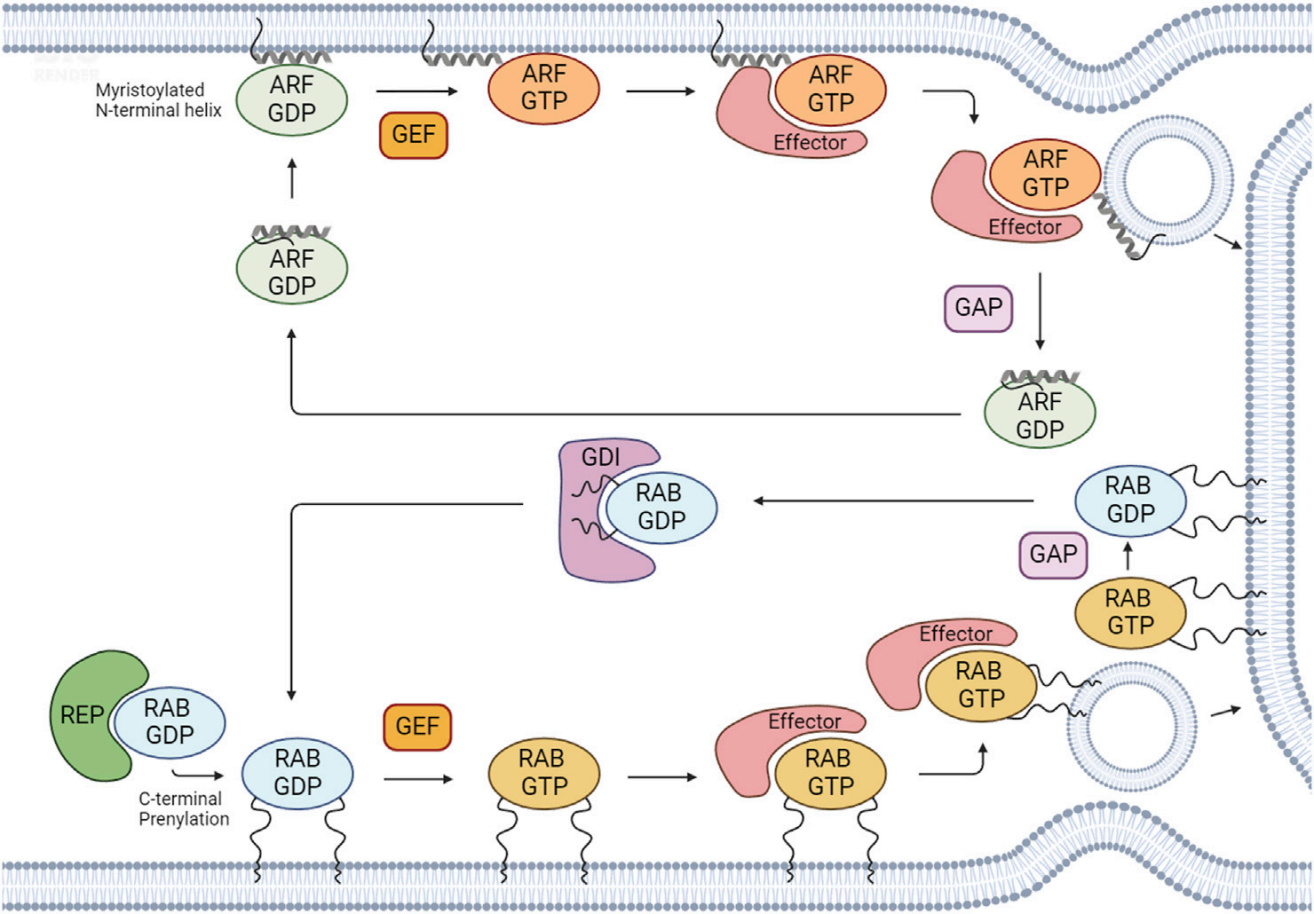
Regulation of the activation/inactivation cycle and membrane binding of RABs and ARFs. After RAB proteins are synthesized, they associate with cytosolic RAB escort proteins (REPs) to form a stable complex. To associate with membranes, RABs and ARFs undergo prenylation and myristoylation, respectively, which are the most common post-translational modifications for these GTPases. RABs and ARFs cycle between an active GTP-bound state and an inactive GDP-bound state. Active GTP-bound forms bind to effectors to regulate several cellular processes. The GDP/GTP cycle is regulated by guanine nucleotide exchange factors (GEFs) and GTPase-activating proteins (GAPs). GEFs catalyze the exchange of GDP for GTP and GAPs promote the hydrolysis of GTP to GDP. RAB GDP dissociation inhibitors (GDIs) can maintain RAB GTPases in an inactive GDP-bound state by sequestering the proteins in their GDP-bound form and preventing their activation. Created with BioRender.

Membrane trafficking is a highly regulated intracellular communication system that allows the specific transport of lipids, proteins and other cargoes between different membrane-bound compartments, ensuring normal cell and tissue homeostasis (Zhao and Zhang, 2020). This system involves the formation of vesicles that transport selected cargoes between donor and acceptor compartments. Membrane trafficking can be divided in 5 steps, namely: vesicle budding, transport, tethering, docking, and fusion (Mima, 2018). Members of the RAS superfamily of small guanosine triphosphate (GTP)-binding proteins (GTPases) play key roles in membrane trafficking. This superfamily comprises five different families: RAS oncoproteins, RAS homologous (RHO) proteins, RAS-related in brain (RAB), RAS-like nuclear proteins (RAN) and ADP-ribosylation factor (ARF) proteins (Wennerberg et al., 2005). RAS GTPases function as molecular switches, alternating between an active state, when bound to GTP, and an inactive state, when bound to guanosine diphosphate (GDP) (Arrazola Sastre et al., 2021) (Figure 1). When active, these proteins recruit effectors, allowing them to perform downstream functions.

The RAB family is the largest of the RAS superfamily and its members are master regulators of all steps of membrane trafficking. These proteins are evolutionarily conserved and found in several organisms, from yeast to humans. To date, more than 60 RABs have been identified in the human genome, divided into 44 subfamilies (Jin et al., 2021). Importantly, several RAB subfamilies comprise distinct isoforms–defined as having >70% of homology–that can perform (partially) redundant functions (Pereira-Leal and Seabra, 2000). RAB proteins have several highly conserved regions that are also present in other members of the RAS superfamily. This is the case of the switch I and switch II regions, which change conformation upon GTP binding, and interact with effector proteins. Additionally, RABs possess a hypervariable C-terminal motif that displays one of the following combinations of aminoacids: XXXCC, XXCCX, XCCXX, CCXXX, XXCXC and XCXXX (where X is any aminoacid) (Pylypenko et al., 2018). The cysteines are subjected to a post-translation modification named prenylation, which consists in the addition of hydrophobic geranylgeranyl groups, and is essential for membrane binding (Leung et al., 2006) (Figure 1). RABs assist in vesicle formation/budding, allow the transport of cargoes within the cell by interacting with motor proteins, and regulate tethering, docking and fusion of vesicles with acceptor compartments.

The ARF family of small GTPases includes ca. 30 proteins in mammals, which are also key regulators of membrane trafficking. This family includes 6 ARFs (5 in humans, since we lack ARF2), 22 ARF-like (ARL) proteins, two secretion-associated RAS-related (SAR), and the tripartite motif-containing protein 23 (TRIM23) protein (Reiner and Lundquist, 2018; Sztul et al., 2019). ARFs1-6 are highly conserved in structure and sequence. Moreover, these proteins are classified into three types, based on their sequence: type I (ARF1-ARF3), which share more than 96% sequence identity; type II (ARF4 and ARF5), which share 90% sequence identity; and type III (ARF6), which shares more than 65% sequence identity with type I and II ARFs. ARF1-5 localize to the Golgi, while ARF6 localizes to the plasma membrane (PM) and endosomes (Donaldson, 2003; Li et al., 2004; Kahn et al., 2006). SAR1A and SAR1B have high homology, sharing around 90% of sequence identity, and localize to the endoplasmic reticulum (ER). These proteins regulate the budding of vesicles coated with coat protein complex II (COPII) and are involved in ER-to-Golgi trafficking (Saito et al., 2017; Sztul et al., 2019). TRIM23 is implicated in antiviral defense, through the regulation of autophagy and adipocyte differentiation (Arimoto et al., 2010; Watanabe et al., 2015). ARLs are more divergent than ARFs or SARS, sharing between 40% and 60% of identity among them (Sztul et al., 2019). Furthermore, these proteins localize to several compartments within the cell and participate in multiple cellular processes, including cargo sorting at the Golgi, lysosome positioning, cilia function, cytoskeleton dynamics, among others (Marwaha et al., 2019). Members of the ARF family can be further subdivided into different paralogs (*e.g.*, ARL13A and ARL13B) that arose from a common ancestor (Kahn et al., 2014). One feature that distinguishes the ARF family from the other families of RAS small GTPases, including RABs, is the presence of an N-terminal extension of around 14 amino acids that is post-translationally modified to allow membrane binding. Moreover, while ARFs are all myristoylated, ARLs can be myristoylated, palmitoylated or acetylated (Donaldson and Jackson, 2011; Sztul et al., 2019).

The activation/inactivation of RABs and ARFs is tightly regulated to ensure the occurrence of specific cellular processes at precise times and locations. The cycling between GTP- and GDP-bound states is controlled by guanine nucleotide exchange factors (GEFs) and GTPase-activating proteins (GAPs). GEFs catalyze the exchange of GDP for GTP, activating these proteins. Conversely, GAPs catalyze the hydrolysis of GTP to GDP, which leads to their inactivation (Figure 1).

The activity of RAB GTPases is intrinsically associated with their membrane binding capacity. After RABs are synthesized, they associate with cytosolic RAB escort proteins (REP1 and 2), forming a stable complex (Alory and Balch, 2003). Subsequently, REPs present RAB proteins to the enzyme RAB geranylgeranyl transferase (RABGGTase), which catalyzes their prenylation. Moreover, RABs are further recognized by RAB GDP -dissociation inhibitors (GDIs). GDIs assist in the dissociation of geranylgeranylated RABs from membranes, allowing their stabilization in the cytosol (Figure 1).

In the case of ARF family proteins, they need to be recruited to membranes to be activated. Indeed, ARFs are recruited to membranes via an N-terminal amphipathic helix in the GDP-bound state. After GEF-mediated GDP/GTP exchange, a conformational rearrangement allows the insertion of the lipid group into the lipid bilayer, stabilizing these proteins on intracellular membranes (Sztul et al., 2019; Li and Guo, 2022) (Figure 1).

As regulators of all membrane trafficking steps, RAB and ARF family proteins are essential for the maintenance of cellular homeostasis. Cargoes transported by this system are determinant for several biological processes such as migration, invasion, metabolism and autophagy. Therefore, it is not surprising that the subversion of these mechanisms by tumor cells plays a key role in BC progression (Casalou et al., 2016; Casalou et al., 2020; Jin et al., 2021; Chen et al., 2022). Additionally, mutations and amplifications and/or post-translational modification changes of ARFs and RABs often lead to the dysregulation of their expression and activity (Casalou et al., 2020; Matos, 2021). These have increasingly been recognized as having an important role in BC. Herein, we review the function in membrane trafficking of the members of the RAB and ARF families implicated in BC, and discuss how they are modulated (Table 1). We divided the proteins reviewed according to the pathways of membrane trafficking they regulate, namely secretion/exocytosis; endocytosis/recycling; lysosomes/autophagy; cytoskeleton dynamics; ciliary functions; and others. We also discuss how the regulation of ARFs and RABs by GEFs and GAPs contributes to BC cell proliferation, migration and invasion. Our main goal is to highlight the relevance of the subversion of membrane trafficking regulators in BC progression, and propose how they could be used as therapeutic targets or prognostic markers.

**TABLE 1:** Expression of ARF and RAB family proteins in human breast cancer samples and/or cell lines.

Membrane trafficking pathways	RABs/ARFs	Subcellular localization	Up/Downregulated in breast cancer	References
Secretion/Exocytosis	RAB2A	ER, Golgi, bidirectional trafficking	Upregulated	Kajiho et al. (2016)
	RAB3B	Synaptic vesicles and secretory vesicles, Golgi (non-secretory cells)	Upregulated	Zhang et al. (2020)
	RAB3D	Synaptic vesicles and secretory vesicles, Golgi (non-secretory cells)	Upregulated	Yang et al. (2015)
	RAB8A	TGN, PM	Upregulated	Bravo-Cordero et al. (2007), Liu et al. (2022)
	RAB27A	Secretory vesicles	Upregulated	Wang et al. (2008), Bobrie et al. (2012)
	RAB27B	Secretory vesicles	Upregulated	Hendrix et al. (2010), Zhang et al. (2012)
	RAB40B	Golgi	Upregulated	Jacob et al. (2016)
	ARF1	Cytosol, Golgi	Upregulated	Schlienger et al. (2015), Luchsinger et al. (2018), Qin et al. (2021)
	ARF3	Cytosol, Golgi	Upregulated	Huang et al. (2019), Zhang et al. (2019)
	ARF4	Cytosol, Golgi, endosomes	Upregulated	Jang et al. (2012), Howley et al. (2018)
	ARFRP1	TGN	ND	Gao et al. (2022)
	ARL4C	Cytosol, PM	Downregulated	Li et al. (2020)
Endocytosis/Recycling	RAB5A	EEs	Upregulated	Frittoli et al. (2014)
	RAB13	TGN, REs, LEs PM	ND	Sahgal et al. (2019)
	RAB11A	ERC, TGN	Upregulated	Wang et al. (2015)
	RAB11B	ERC, TGN	Upregulated^a^	Howe et al. (2020)
	RAB11C (RAB25)	Apical recycling compartment	Upregulated/Downregulated	Cheng et al. (2010), Mitra et al. (2016)
	RAB21	EEs	ND	Pellinen et al. (2006)
	RAB34	Endosomes, lysosomes	Upregulated	Sun et al. (2018)
	RAB35	Endosomes, PM	Upregulated/Downregulated	Allaire et al. (2013), Zhu et al. (2013), Deng et al. (2016)
	ARF6	PM, endosomes, RE, cortical actin	Upregulated	Hashimoto et al. (2004), Morishige et al. (2008), Huang et al. (2022)
Lysosomes/Autophagy	RAB26	EEs, LEs, lysosomes, secretory granules	Downregulated	Liu et al. (2021)
	ARL8A	Lysosomes	Upregulated	Kothari et al. (2021)
	ARL8B	Lysosomes	Upregulated	Wu et al. (2020), Kothari et al. (2021)
Cytoskeleton dynamics	ARL2	Cytosol, mitochondria	Downregulated	Beghin et al. (2007), Beghin et al. (2008), Beghin et al. (2009)
Ciliary trafficking	ARL13B	Cilia, actin, EEs, REs, CDRs	Upregulated	Casalou et al. (2019)
Others	ARL11	Nucleus, cytosol, cortical actin	ND	Frank et al. (2006), Yendamuri et al. (2008)

^a^ RAB11B is upregulated in brain metastases.

RAB, RAS-related in brain; ARF, ADP-ribosylation factor; ARL, ARF-like; ND, not determined; CDRs, circular dorsal ruffles; EEs, early endosomes; ER, endoplasmic reticulum; ERC, endocytic recycling compartment; LEs, late endosomes; PM, plasma membrane; REs, recycling endosomes; TGN, *trans*-Golgi network.

## 2 Secretion/exocytosis

### 2.1 RAB2A

RAB2 family comprises two isoforms (RAB2A and RAB2B) that localize to the Golgi, regulating bidirectional trafficking between the ER and the Golgi (Goud et al., 2018). High expression of RAB2A was detected in BC, compared to adjacent normal mammary tissue (Kajiho et al., 2016). Moreover, RAB2A is significantly associated with poor prognosis markers (Kajiho et al., 2016). Additionally, RAB2A was shown to mediate the exocytosis of membrane type 1-matrix metalloproteinase (MT1-MMP), an essential MMP for extracellular matrix (ECM) remodeling and BC cell invasion. Furthermore, RAB2A controls the trafficking of E-cadherin from the Golgi to the PM, ultimately regulating cell compaction, junctional stability, and tumor invasiveness (Kajiho et al., 2016; Kajiho et al., 2017).

### 2.2 RAB3

RAB3 subfamily comprises four functionally redundant isoforms: RAB3A, RAB3B, RAB3C and RAB3D. They share a high degree of protein sequence homology (∼80%) and similar subcellular localization, namely in synaptic and secretory vesicles (Schlüter et al., 2002). RAB3A, RAB3B, RAB3C are primarily expressed in neuronal cells, while RAB3D is mostly observed in non-neuronal secretory cells, such as pancreas and mast cells (Millar et al., 2002; Riedel et al., 2002; Schlüter et al., 2002). Although the precise functions of the different RAB3 isoforms are not entirely established, they are involved in synaptic vesicle exocytosis (Schlüter et al., 2006) and secretory granule maturation (Kögel et al., 2013). Moreover, RAB3A and RAB3D were found to be required for docking and fusion of lysosomes and secretory vesicles, respectively, during regulated secretion in non-neuronal cells (Millar et al., 2002; Encarnação et al., 2016). Interestingly, RAB3A, RAB3B and RAB3D were shown to promote breast, colon, esophagus, melanoma, osteosarcoma and glioma tumor progression by increasing cell proliferation, migration, and invasion (Raffaniello, 2021). Additionally, RAB3D expression levels are higher in BC cells and positively correlate with tumor stage (Yang et al., 2015). Furthermore, RAB3D was found to regulate epithelial-to-mesenchymal transition (EMT) and promote BC tumor cell motility, invasion and metastasis through the activation of the AKT/GSK-3β/SNAIL signaling pathway (Yang et al., 2015). Finally, RAB3B expression is upregulated in aggressive luminal B BC cells in response to the amplification of the inositol-requiring enzyme 1 (*IRE-1*) gene, which acts as an oncogenic factor to repress a subset of tumor suppressor microRNAs (miRs) via regulated IRE1-dependent decay (Zhang et al., 2020).

### 2.3 RAB8

RAB8 has two isoforms, RAB8A and RAB8B, which mediate the trafficking from the *trans*-Golgi network (TGN) to the PM, controlling the apical/basolateral transport of proteins in epithelial cells (Sato et al., 2007; Stenmark, 2009). In BC, RAB8 regulates the exocytosis of MT1-MMP, which promotes collagen degradation and cell invasion (Bravo-Cordero et al., 2007). Recently, Liu et al. showed that RAB8A is upregulated in BC (Liu et al., 2022). Moreover, RAB8A promotes the surface expression of tropomyosin-related kinase B (TRKB), which leads to the proliferation, migration and invasion of BC cells, through activation of the AKT and extracellular signal-regulated kinase (ERK) 1/2 signaling pathways (Liu et al., 2022).

### 2.4 RAB27

The two isoforms of RAB27—RAB27A and RAB27B–are involved in regulated secretion. These have 71% of homology and recruit the same effector proteins. However, the underlying mechanisms by which they are involved in BC are different (Li et al., 2018). RAB27 isoforms are associated with lysosome-related organelles (LROs) and control exosome secretion in BC (Li et al., 2018). Indeed, RAB27A has been associated with the upregulation of exosome secretion by enhancing the secretion of insulin growth factor-II (IGF-II) in BC subtypes TNBC and HER2+ (Wang et al., 2008). RAB27B is upregulated in ER-positive BC and associated with an increased secretion of mesenchymal proteins, like vimentin and fibronectin, to the extracellular milieu (Bobrie et al., 2012; Zhang et al., 2012). Therefore, RAB27B upregulation is associated with poor prognosis in BC (Hendrix et al., 2010; Zhang et al., 2012).

### 2.5 RAB40

The RAB40 subfamily contains four isoforms (RAB40A, RAB40AL, RAB40B, and RAB40C), which are enriched in the Golgi (Stenmark, 2009; Duan et al., 2021). In BC, RAB40B was shown to interact with tyrosine kinase substrate with 5 SH3 domains (TKS5), an adaptor protein that acts as a scaffold, bringing membrane and intracellular elements close to invadopodia (Jacob et al., 2013; Jacob et al., 2016). This interaction is required to target MMP-2- and MMP-9-containing vesicles to invadopodia, promoting ECM remodeling during BC progression (Jacob et al., 2013; Jacob et al., 2016). Moreover, the same authors found RAB40B to be highly expressed in more aggressive cancers, as well as in basal BC subtype. Additionally, RAB40C was found to regulate focal adhesion number, size, and distribution in migrating BC cells (Han et al., 2022).

### 2.6 ARF1

ARF1 localizes to the Golgi, where it regulates the function and structure of this compartment (Adarska et al., 2021). In BC, ARF1 was found to be highly expressed and fundamental for epidermal growth factor (EGF)-mediated phosphorylation of focal adhesion kinase (FAK) and Src, regulating BC cell proliferation and adhesion (Schlienger et al., 2015). The same group performed *in vivo* studies and showed that ARF1 promotes primary tumor growth and the formation of metastases (Schlienger et al., 2016). Indeed, in non-invasive MCF-7 BC cells, ARF1 overexpression leads to the formation of lung metastases. In the same study, the authors found a link between ARF1 and the regulation of several pathways that are involved in EMT (E-cadherin/β-catenin, RAS, ERK1/2 and PI3K/AKT), as well as the upregulation of EMT markers SLUG and SNAIL. Moreover, ARF-1-expressing cells lose their epithelial features and acquire a more mesenchymal shape. Finally, ARF1 overexpression in MCF-7 cells leads to the activation of MMP-2 via FAK, contributing to the role of ARF1 in BC invasion (Schlienger et al., 2016). Additionally, ARF1 disruption sensitizes TNBC (MDA-MB-231) cells to the anti-tumor drugs actinomycin D and vinblastine (Luchsinger et al., 2018). Furthermore, it was shown that the recruitment of ARF1 and the ARF GAP ARAP1 to circular dorsal ruffles (CDRs) promotes shear stress-induced BC cell migration (Qin et al., 2021).

### 2.7 ARF3

ARF3 regulates the recruitment of coat complexes to the Golgi apparatus and promotes the activation of phospholipase D (PLD) and phosphatidyl-kinases (PI-kinases) (Sztul et al., 2019). In BC, it was shown that the clinical stage positively correlates with ARF3 expression, which is upregulated in 92.8% of malignant cases (Huang et al., 2019). Furthermore, ARF3 mRNA and protein expression levels were found upregulated in BC cell lines and tissues (Huang et al., 2019). It was also observed that ARF3 overexpression promotes BC cell proliferation, through the regulation of the G1/S cell cycle transition (Huang et al., 2019). A recent study, based on integrated analysis of microarray profile datasets, revealed that ARF3 is a candidate gene involved in the progression of pregnancy-associated BC (Zhang et al., 2019).

### 2.8 ARF4

As described for ARF1 and ARF3, ARF4 also localizes to the Golgi, being required for the recruitment of coat proteins and the retrieval of ER-resident proteins (Pennauer et al., 2021). ARF4, as well as the ER-Golgi trafficking regulators COPI subunit β1 (COPB1) and USO1, were found to be upregulated in BC patient samples (Howley et al., 2018). Moreover, it was reported that ARF4, COPB1, and USO1 regulate BC cell growth and invasion by mediating the retrograde transport of proteins from the Golgi to the ER via COPI-coated vesicles (Howley et al., 2018). ARF4 was also shown to promote BC cell migration in response to phorbol-12-myristate 13-acetate (PMA), a known gene expression inducer (Jang et al., 2012).

### 2.9 ARFRP1

ARF-related protein 1 (ARFRP1) localizes to the TGN and mediates the trafficking/sorting of various cargoes (e.g., glucose transporters and E-cadherin) (Hesse et al., 2010). Furthermore, recent studies have shown that ARFRP1 regulates the recruitment of tethering factors to the TGN, upstream of ARL1 and ARL5 (Ishida and Bonifacino, 2019). In particular, ARFRP1 is involved in the recruitment of golgins, which are dependent on ARL1, and Golgi-associated retrograde protein (GARP), which is dependent on ARL5 (Ishida and Bonifacino, 2019). A recent proteome-based study proposed that ARFRP1 regulates the radioresistance of BC cells (Gao et al., 2022). Moreover, the authors found that ARFRP1 silencing leads to an increase in radiation resistance of both MCF-7 (luminal A) and MDA-MB-231 (TNBC) BC cell lines (Gao et al., 2022).

### 2.10 ARL4C

ARL4 has three paralogs (ARL4A, ARL4C and ARL4D) that localize to the PM. ARL4C was also found to localize to the cytosol and the nucleus, and interact with α-tubulin. Additionally, ARL4C regulates transferrin receptor transport from early endosomes (EEs) to recycling endosomes (REs) (Wei et al., 2009). Moreover, ARL4C was shown to be downregulated in BC samples. Furthermore, the low levels of ARL4C in BC correlate with those of the activated transcription factor 3 (ATF3), which is also downregulated in BC (Li et al., 2020). These authors also found that ATF3 promotes the transcription of ARL4C, which acts as a negative regulator of breast tumor progression. Indeed, the overexpression of ARL4C was shown to decrease BC cell proliferation, migration, and invasion, leading to cell cycle arrest (Li et al., 2020).

## 3 Endocytosis/recycling

### 3.1 RAB5A

RAB5 has three distinct isoforms (RAB5A, RAB5B, RAB5C) that share more than 90% of sequence identity (Chen et al., 2009). RAB5 plays a key role in endocytosis regulation, mediating the transport and fusion of endocytic vesicles with EEs (Yuan and Song, 2020). In a meta-analysis of five human breast tumor gene expression datasets, RAB5A expression, but not RAB5B or RAB5C, was shown to correlate with poor prognosis (Frittoli et al., 2014). Additionally, RAB5A expression is significantly higher in matched lymph node metastases, compared to their primary tumors. RAB5A transports β3-integrin and MT1-MMP to invadopodia, through a RAB4-dependent pathway, allowing their maturation into competent ECM-degrading structures, which promotes invasion of metastatic BC cells (Frittoli et al., 2014).

### 3.2 RAB13

RAB13 plays a role in both secretory and endocytic recycling pathways. It localizes to the TGN, REs, late endosomes and the PM (Ioannou and McPherson, 2016). Silencing of RAB13 in TNBC MDA-MB-231 cells leads to the intracellular accumulation of active β1-integrin, reducing integrin activity in focal adhesions and impairing cell migration. This suggests that RAB13 plays a role in facilitating the recycling of active β1-integrin to the PM (Sahgal et al., 2019).

### 3.3 RAB21

RAB21 localizes mainly to EEs and the PM, being involved in the early endocytic pathway (Simpson et al., 2004). In BC, RAB21 was shown to regulate the endo/exocytic trafficking of integrins, stimulating MDA-MB-231 TNBC cell adhesion and migration (Pellinen et al., 2006).

### 3.4 RAB11

The RAB11 subfamily encompasses three different isoforms: RAB11A, RAB11B and RAB11C, also known as RAB25. While RAB11A and RAB11B exhibit 90% amino acid identity, RAB11A/B and RAB25 share 60% homology (Kelly et al., 2012). RAB11 proteins localize to various subcellular compartments, including the endocytic recycling compartment (ERC), REs, apical recycling compartment (ARE), and the TGN (Welz et al., 2014). Moreover, they mainly regulate cargo recycling from the ERC to the cell surface, and also participate in exocytic transport from the TGN (Ullrich et al., 1996; Chen et al., 1998; Ren et al., 1998). Recently RAB11A and RAB11B were described by us to be required for Ca^2+^-dependent lysosome exocytosis (Escrevente et al., 2021).

In BC, RAB11 controls the transport of α6β4-integrin from REs and the TGN to the PM, enabling cell invasion under hypoxic conditions (Yoon et al., 2005). The RAB11 isoforms have also been associated with breast carcinogenesis. A miR that targets RAB11A (miR-320a) was found to inhibit proliferation, migration, and invasion of MDA-MB-231 cells *in vitro*, as well as tumor growth in a mouse xenograft model (Wang et al., 2015). Additionally, the authors found that RAB11A mRNA is overexpressed in BC samples, compared to normal adjacent tissues (Wang et al., 2015). In a subsequent study, miR-452 was found to be a tumor suppressor gene that also inhibits BC cell migration and invasion by targeting RAB11A (Li et al., 2017b). Notably, RAB11A is upregulated in DCIS, a non-obligatory precursor of invasive BC, when compared to adjacent normal tissues (Palmieri et al., 2006). In the same study, the overexpression of a dominant-negative RAB11A mutant (S25N) was observed to lead to decreased EGF receptor (EGFR) recycling and cell proliferation in MCF10A human breast epithelial cell line (Palmieri et al., 2006).

The role of RAB11B in BC remains relatively unexplored. However, a recent study highlighted the importance of RAB11B in the adaptation of BC metastases to the brain microenvironment. Specifically, RAB11B regulates the recycling of β1-integrin, enabling effective interaction between BC cells and the brain ECM (Howe et al., 2020).

Interestingly, RAB25 plays a dual role in BC, functioning as a tumor promoter in luminal cancers, and as a tumor inhibitor in TNBC. Indeed, expression of RAB25 positively correlates with ER- and PR-positive BCs and lymphatic metastasis (Yin et al., 2012). Moreover, it was shown that RAB25 increases proliferation and migration of luminal B BC cell lines (Mitra et al., 2016). On the other hand, RAB25 expression is lost in hormone receptor-negative BC compared to matched normal tissues and overexpression of this isoform reduces the proliferation of MDA-MB-231 cells (Cheng et al., 2010).

### 3.5 RAB34

RAB34 regulates endosomal trafficking, autophagy and lysosome maturation, by mediating the distribution of lysosomes to the perinuclear region (Starling et al., 2016). Moreover, it facilitates the internalization of receptors, transport of endocytic vesicles to peri-Golgi regions, and regulates the fusion of phagosomes with lysosomes (Kasmapour et al., 2012). In BC, RAB34 overexpression was associated with increased tumor invasiveness, migration and metastatic potential, as well as the recycling of β3-integrin (Wang and Hong, 2005; Sun et al., 2018).

### 3.6 RAB35

RAB35 localizes to REs and the PM of different cell types, and plays a role in several cellular functions, including endocytic recycling, cytokinesis, cell polarity, exosome release, immunity, lipid homeostasis, and phagocytosis (Klinkert and Echard, 2016). Therefore, it is not surprising that RAB35 can exert an important role in different types of cancer by controlling several aspects of cancer progression, such as cell migration, proliferation and survival (Shaughnessy and Echard, 2018). In MCF-7 luminal A BC cells, RAB35 activation by Wnt5a promotes cell migration via the DVL2/RAB35/RAC1 signaling pathway (Zhu et al., 2013). EGF-activated RAB35 can also lead to a more invasive phenotype in BC cells. In its active form, RAB35 binds to microtubule-associated monooxygenase, calponin and LIM domain containing-1 (MICAL-1) and promotes its activation. MICAL-1 activation increases reactive oxygen species (ROS) generation and AKT phosphorylation, leading to a more invasive phenotype (Deng et al., 2016). Additionally, RAB35 expression was found to be downregulated in highly invasive BC tumors, where ARF6 is hyperactivated (Allaire et al., 2013). Enhanced ARF6 activation leads to integrin and EGFR recycling to the cell surface, promoting cell migration (Allaire et al., 2013). Finally, it was shown that some RAB35 pathogenic somatic mutations (G18V, A29V and F45L) in BC can activate this protein and confer it oncogenic properties (Shaughnessy and Echard, 2018).

### 3.7 ARF6

ARF6 is mainly localized at the cell periphery, where it regulates endocytic recycling and cortical actin dynamics. Moreover, this small GTPase is involved in the regulation of cell division (Sztul et al., 2019). ARF6 has been extensively studied in cancer and it is known to regulate cancer cell growth, angiogenesis, invasion, and the formation of metastases (Li R. et al., 2017). Moreover, high ARF6 expression and the activation of downstream signaling pathways were associated with poor overall survival of BC patients (Hashimoto et al., 2004). It was also shown that ARF6 has a role in BC cell invasion, as it was found to localize to invadopodia and regulate the activity of these actin-rich structures, which promote ECM degradation in tumors (Hashimoto et al., 2004). Indeed, a study using BC cell lines with different invasive capacities showed a correlation between ARF6 protein levels and BC cell invasiveness (Hashimoto et al., 2004). The same authors also observed that ARF6 silencing decreases the invasion capacity of BC cells and regulates EGFR signaling (Morishige et al., 2008). A recent study showed that ARF6 targets palmitoylated EGFR to promote its trafficking from the Golgi to the PM, through the recruitment of the exocyst tethering complex (Guo et al., 2022). As mentioned, matrix remodeling/degradation is a key feature of BC cell invasion. ARF6 activity and its effectors JIP3 and JIP4 have been associated with MDA-MB-231 cell invasion through invadopodia-mediated mechanisms (Marchesin et al., 2015). In particular, the authors found that the binding of active ARF6 to JIP3/JIP4 regulates the trafficking and exocytosis of MT1-MMP, a key regulator of invadopodia function (Tanaka and Sakamoto, 2023). Furthermore, this study showed that the regulation of MT1-MMP trafficking occurs via the recruitment of motor proteins (dynactin-dynein and kinesin-1) by JIP3/JIP4 (Marchesin et al., 2015). Recently, the role of ARF6 was associated with the tumor microenvironment. Specifically, the chemokine (C-C motif) ligand 18 (CCL18), which is mainly produced by tumor-associated macrophages and has been linked to the formation of metastases in BC, was found to increase the expression of ARF6 and the phosphorylated form of its downstream effector AMAP1, an ARF GAP, in MCF-7 cells (Huang et al., 2022). In the same study, the authors observed that CCL18 increases the levels of miR-760 in exosomes, which activates an ARF6/Src/PI3K/AKT signaling cascade and induces BC cell proliferation, migration, invasion, and drug resistance.

## 4 Lysosomes/autophagy

### 4.1 RAB26

RAB26 is involved in several processes, including exocrine granule maturation, amylase release from parotid acinar cells and lysosome clustering in the perinuclear region (Nashida et al., 2006; Tian et al., 2010; Li et al., 2012). Moreover, RAB26 increases the integrity of adherens junctions in acute lung injury and regulates the trafficking of cell surface receptors such as α2-adrenergic receptor (Li et al., 2012; Dong et al., 2018). In BC, RAB26 acts as a tumor suppressor gene by reducing focal adhesion association of Src kinase and inducing autophagic degradation of phosphorylated Src, which results in the inhibition of migration and invasion of BC cells (Liu et al., 2021). Furthermore, BC datasets showed that higher RAB26 expression is associated with a significantly higher overall survival (Liu et al., 2021).

### 4.2 ARL8

ARL8 has two paralogs–ARL8A and ARL8B–both localized to lysosomes. Whereas the function of ARL8A is still poorly understood, ARL8B is well studied and known to regulate the kinesin-dependent anterograde movement (towards the cell periphery) of lysosomes (Rosa-Ferreira and Munro, 2011; Khatter et al., 2015). Interestingly, two recent reports showed evidence that ARL8 also regulates long-range endolysosomal retrograde movement (towards the perinuclear region) (Keren-Kaplan et al., 2022; Kumar et al., 2022). ARL8A and ARL8B were found to be upregulated in both luminal A and TNBC cell lines, upon silencing of the Ca^2+^-binding protein TBC1 domain family member 9 (TBC1D9, a RAB GAP), which is associated with an impairment of TNBC progression (Kothari et al., 2021). A recent study showed evidence that the movement of lysosomes towards the cell periphery promotes the invasion of radiation-surviving BC cells *in vitro*, as well as tumor metastasis *in vivo* (Wu et al., 2020). In particular, the recruitment by the biogenesis of lysosome-related organelles complex 1 (BLOC-1)-related complex (BORC) and activation of ARL8B leads to binding to the effector Sif-A and kinesin-interacting protein (SKIP), which promotes lysosome anterograde movement (Wu et al., 2020). At the cell periphery, lysosomes can be exocytosed and release ECM-degrading proteases, leading to BC cell invasion (Machado et al., 2015; Wu et al., 2020).

## 5 Cytoskeleton dynamics

### 5.1 ARL2

ARL2 is highly conserved, and it was found to localize to the cytosol and mitochondria (Sztul et al., 2019). Moreover, this small GTPase was shown to regulate the α/β-tubulin biogenesis and microtubule dynamics, as well as mitochondria motility, fusion, and ATP levels (Bhamidipati et al., 2000; Newman et al., 2014; Francis et al., 2017; Newman et al., 2017). ARL2 was also shown to regulate α/β-tubulin polymerization in MCF-7 cells. Furthermore, it was also observed that a lower expression of ARL2 leads to enhanced resistance to cytotoxic agents (Beghin et al., 2007; Beghin et al., 2008). This mechanism is regulated by protein phosphatase 2A (PP2A), which fails to dephosphorylate p53 when ARL2 expression is low (Beghin et al., 2008). The same group also used orthotopic mouse models to show that ARL2-depleted BC cells have an enhanced clonogenic potential, less contact inhibition, and proliferation, as well as impaired tumor growth (Beghin et al., 2009).

## 6 Cilliary trafficking

### 6.1 ARL13B

ARL13 presents two paralogs (ARL13A and ARL13B) that only share around 43% sequence homology (Marwaha et al., 2019). ARL13B is a well-established regulator of cilia structure and function (Seixas et al., 2016; Revenkova et al., 2018). Moreover, our group described that the interaction of ARL13B with the actin cytoskeleton, mediated by its effector non-muscle myosin IIA (NMIIA), promotes the formation of CDRs and, consequently, cell migration (Casalou et al., 2014). Furthermore, we showed that ARL13B plays a role in BC progression, through a mechanism likely independent of cilia (Casalou et al., 2019). Specifically, we found that the silencing of ARL13B leads to an impairment in BC cell migration and invasion *in vitro*, as well as tumor growth and metastasis *in vivo*. Moreover, we gathered evidence to support that ARL13B promotes BC cell migration and invasion through the regulation of integrin-dependent signaling and cell-ECM adhesion (Casalou et al., 2019). The results obatined in this study suggest that ARL13B interacts with β3-integrin, regulates the formation of stress fibers and the size of focal adhesions, which results in the modulation of the cell-ECM adhesion and cell motility (Casalou et al., 2019).

## 7 Other functions

### 7.1 ARL11

ARL11, which is also named ADP-ribosylation factor-like tumor suppressor gene-1 (ARLTS1), was initially described as a tumor suppressor (Calin et al., 2005). Yet, little is known about its function(s) and localization. Nevertheless, in a recent study, it was observed that ARL11 localizes to the nucleus, cytosol, and cortical actin (Arya et al., 2018). These authors also found that ARL11 is essential for liposaccharide (LPS)-induced macrophage activation by interacting with phosphorylated ERK1/2 on actin structures (Arya et al., 2018). Different variants of ARL11 have been linked to familial and sporadic cases of cancer. In this regard, the mutations W149Stop and C148R are the best characterized (Yendamuri et al., 2008). The former is a nonsense mutation that results in the production of a truncated protein that is unable to bind GTP, which leads to a decrease in apoptosis (Petrocca et al., 2006). Furthermore, both mutations were associated with familial cases of BC (Frank et al., 2006; Yendamuri et al., 2008).

## 8 RAB and ARF GEFs/GAPs in BC progression

GEFs and GAPs regulate the activity of RAB and ARF proteins, by promoting their activation or inactivation, respectively (Arrazola Sastre et al., 2021). Consequently, the dysregulated expression of GAPs/GEFs or altered interactions between GEFs/GAPs and RAB/ARF proteins can play a pivotal role in BC progression. For instance, it was shown that DENND2B, a GEF for RAB13, enhances RAB13-mediated migration and invasion of MDA-MB-231 cells both *in vitro* and *in vivo* (Ioannou et al., 2015). Moreover, Morishige et al. showed that GEP100/BRAG2, an ARF6 GEF, is expressed in 70% of primary breast ductal carcinomas and contributes to the invasive behavior of MDA-MB-231 BC cells (Morishige et al., 2008). Specifically, the authors found that GEP100 binds to tyrosine-phosphorylated EGFR to induce ARF6 activation, which promotes the invasiveness of BC cells (Morishige et al., 2008). Another study demonstrated that GEP100 mediates EGF-induced cell invasion, through a ARF6/ERK/uPAR signaling cascade (Hu et al., 2013). Furthermore, the ARF6 GEF EFA6B enables the transport of EGFR to the PM, which promotes the progression of EGFR-dependent tumors (Guo et al., 2022).

Similar to GEFs, RAB and ARF GAPs also play a role in BC progression. For instance, the RAB5 GAP USP6NL, which regulates endocytosis and signal transduction, is overexpressed in BC (Avanzato et al., 2018). The authors also found that the depletion of USP6NL in BC cells results in a decrease in EGFR/AKT levels, GLUT1 degradation, and consequently, a reduction in cell proliferation. Moreover, the depletion of RAB5 phenocopies the effects of USP6NL, suggesting that RAB5 inactivation by USP6NL is the mechanism involved in the regulation of BC cell metabolism and growth (Avanzato et al., 2018). Furthermore, the balance between ARF-GDP and ARF-GTP levels was found to be essential for MT1-MMP trafficking and consequent invasion of BC cells (Loskutov et al., 2015). This study highlighted the role of a scaffold protein, NEDD9, which binds specifically to the ARF6 GAP ARAP3. The NEDD9/ARAP3 complex is then targeted to active ARF6 bound to the effector GGA3, promoting GTP hydrolysis and inactivation of this ARF protein (Loskutov et al., 2015).

## 9 Discussion

As discussed, membrane trafficking regulators from the RAB and ARF families are abnormally expressed in BC and several studies linked the role of these small GTPases to BC progression. Therefore, the development of new therapeutic tools that target RABs/ARFs could be a promising strategy in BC treatment. However, modulation of these proteins poses some challenges. Indeed, they have very conserved regulatory roles, are expressed in many cells and tissues and share structural features. Yet, there is an increasing effort to find suitable strategies to modulate RAB/ARF activity and expression. First, the development of nucleotide-based competitive inhibitors is being explored to block RAB/ARF activity. For example, CID1067700 is a direct competitor of nucleotide binding and was originally developed to target RAB7 (Hong et al., 2015). By maintaining the RAB in an inactive conformation, this molecule effectively hinders the interaction between the GTPase and downstream effectors. Besides targeting RAB7, CID1067700 also shows inhibitory effects to other small GTPases, including CDC42, RAS and RAB2 (Agola et al., 2012; Hong et al., 2015). Importantly, pan-small GTPase inhibitors may serve as a template for designing more specific drugs in the future. Also, recent studies showed that demethylzeylasteral (DMZ) inhibits ARF1 activity by competing with GTP and impairing ARF1 activation (Chang et al., 2022). Moreover, treatment of 4T1 BC cells with DMZ, leads to decreased proliferation and reduced levels of ARF1-GTP (Chang et al., 2022). A dual ARF and RAS inhibitor, Rasarfin, was described to inhibit receptor internalization, by targeting ARF6, and signaling, by targeting RAS. Rasarfin was also tested in MDA-MB-231 cells and shown to promote a reduction in cell metabolism and both RAS and ARF6 activities (Giubilaro et al., 2021). There is also evidence that non-competitive inhibitors can be used to target ARF activity. In particular, it was found that the antibiotic chlortetracycline (CTC), which belongs to the tetracycline family, inhibits ARF6 activity by preventing GDP/GTP exchange through interaction with Mg^2+^ at the nucleotide-binding site (Macia et al., 2021). Furthermore, treatment of MDA-MB-231 BC cells with CTC impairs collective cell migration and invasion in 3D cultures (Macia et al., 2021).

Targeting the GTPase activity can also be achieved through the modulation of membrane association. RAB proteins must be prenylated to associate with membranes and perform their functions (Leung et al., 2006). Since prenylation is mediated by RABGGTase, there is a growing effort to develop specific RABGGTase inhibitors. Notably, *in vitro* studies have demonstrated promising effects of these inhibitors in reducing human mesothelioma and myeloma cell proliferation, inducing cell cycle arrest and apoptosis, respectively (Roelofs et al., 2006; Okamoto et al., 2014). RAB geranylgeranylation depends on geranylgeranyl pyrophosphate, a product of the mevalonate pathway (Baron and Seabra, 2008; Park et al., 2014). Interestingly, it was recently shown that the inhibition of this pathway, using statins, causes a decrease in BC cell adaptation to the brain microenvironment by suppressing RAB11B activity (Howe et al., 2020). Indeed, non-specific RAB11B inhibition by two lipophilic statins (pitavastatin and simvastatin) impairs the recycling of β1-integrin, subsequently reducing the ability of BC cells to interact with the brain metastatic ECM, effectively suppressing BC brain metastasis (Howe et al., 2020). While all RABs require geranylgeranylation, the authors suggest that inhibiting the mevalonate pathway could potentially be useful in other RAB-mediated clinical scenarios beyond brain metastasis prevention.

Another possible strategy to modulate RAB/ARF activity is the targeting of their GEFs, GAPs or effectors. For example, one can inhibit GEFs or stimulate GAP activity to reduce the levels of active GTP-bound RABs/ARFs that promote cancer progression. Indeed, it was shown that the silencing of DENND2B, a RAB13 GEF, impairs RAB13-mediated migration and invasion of MDA-MB-231 cells *in vitro* and *in vivo* (Ioannou et al., 2015). On the other hand, enhancement of GEF activity or GAP inhibition can increase the levels of GTP-bound RABs/ARFs that act as tumor inhibitors. Additionally, interactions between RABs/ARFs and their GEFs or their downstream effectors can be modulated. One can also target directly downstream effectors by changing their expression levels or blocking their function. Interestingly, a group of stapled peptides that specifically target RAB11:FIP binding interface was shown to effectively disrupt the interaction between various FIPs and both RAB11A and RAB25 (Mitra et al., 2017). These inhibitors, known as RFP14, RFP24, and RFP26, impair RAB11-mediated oncogenic phenotypes, such as migration and proliferation, in BC cell lines (Mitra et al., 2017). In another study, a distinct stapled peptide (StRIP3) was found to exhibit selective binding to RAB8A, resulting in the inhibition of RAB8A-effector interactions *in vitro* (Spiegel et al., 2014). The GEF-mediated activation of ARF1 was shown to be impaired by AMF-26. By targeting the activity of ARF1, AMF-26 was also found to induce Golgi disruption, leading to the regression of BC in BSY-1 xenografts (Ohashi et al., 2012). Interestingly, the ARF GEF inhibitor SecinH3, which impairs the activation of both ARF1 and ARF6, was shown to decrease the growth of BC xenografts and reduce the number of lung metastases (Zhao et al., 2016). Moreover, the inhibitor LM11, which blocks the interaction between ARF1 and the GEF Cytohesin 2/ARNO, was shown to prevent ARF1 activation (Xie et al., 2016). Remarkably, LM11 results in impaired cell migration and invasion of MDA-MB-231 and Hs578T TNBC cells. Furthermore, treatment with LM11 leads to a reduction in the number of BC metastasis in a zebrafish model (Xie et al., 2016). Finally, a study that used artificial membranes containing ARF GTPases and GEFs to discover novel inhibitors, identified Bragsin1 and Bragsin2 as potent inhibitors of the ARF-GEF BRAG2, leading to the impairment of ARF activity (Nawrotek et al., 2019). In the case of ARL13B, the blockade of its effector NMIIA with blebbistatin impairs BC cell invasion (Derycke et al., 2011).

Finally, the use of small interfering RNAs (siRNAs) or miRs to downregulate RAB/ARF expression could also be used as a therapeutic strategy. Notably, miR-320a and miR-452 have been shown to suppress proliferation, migration and invasion of MDA-MB-231 cells, by targeting RAB11A (Wang et al., 2015; Li W. et al., 2017). In addition, ectopic expression of miR-320a inhibits tumor growth in a mouse xenograft model (Wang et al., 2015). Additionally, a recent report showed that miR-139-5p/ARF6 axis can be a promising pathway to target in BC treatment. In this work, the authors showed that the anesthetic drug sevoflurane (SEV), leads to an upregulation of miR-139-5p, which decreases ARF6 expression (Wu et al., 2021). Consequently, the SEV-mediated upregulation of miR-139-5p leads to impaired migration, invasion, and EMT of MCF-7 and MDA-MB-231 BC cells (Wu et al., 2021).

## 10 Conclusion and future perspectives

**FIGURE 2:**
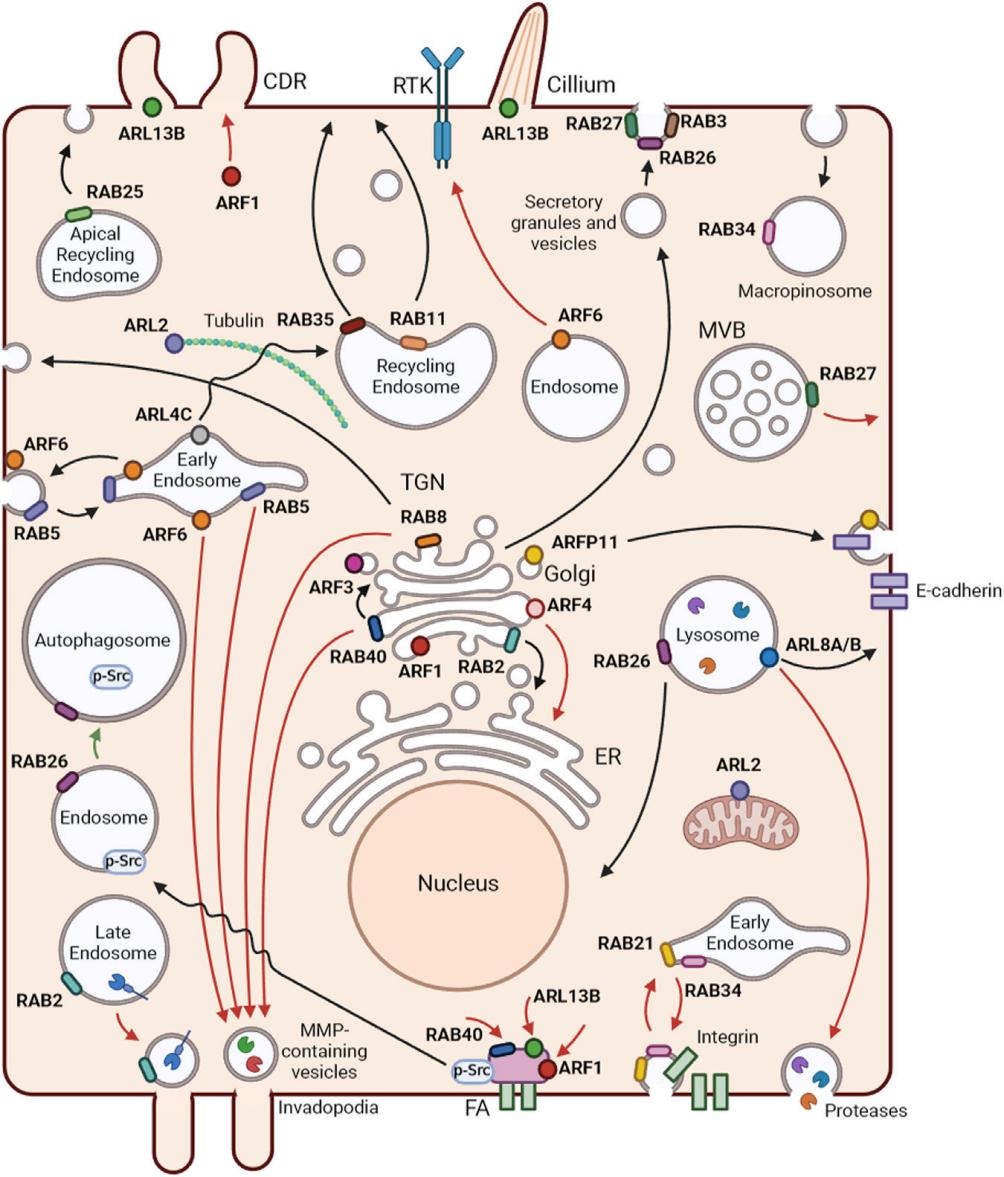
Localization and functions of RAB and ARF GTPases. Representation of membrane trafficking pathways and localization of RABs and ARFs in an epithelial cell. Black arrows represent RAB and ARF functions in non-cancer cells, red arrows show tumor promoter mechanisms and green arrows tumor inhibiting functions, in BC. RAB2 is a key player in Golgi-to-endoplasmic reticulum (ER) trafficking. RAB3 and RAB27 mediate several types of regulated secretion. RAB5 regulates early endosome homotypic fusion. Moreover, RAB5 and RAB34 are involved in macropinocytosis. RAB8 participates in the trafficking from the *trans*-Golgi network (TGN) to the plasma membrane, controlling the apical/basolateral transport of proteins in epithelial cells. RAB11 and RAB35 mediate slow recycling from recycling endosomes. RAB25 controls trafficking from apical recycling endosomes to the apical plasma membrane. RAB26 is involved in exocrine granule maturation and lysosome aggregation in the perinuclear region. RAB40 mediates intra-Golgi trafficking. ARF1 regulates Golgi function and structure. ARF3 and ARF4 control the recruitment of coat proteins to the Golgi. ARFRP1 has been associated with the trafficking of various cargoes, including E-cadherin. ARL2 localizes to the cytosol and mitochondria and regulates α/β-tubulin biogenesis. ARL4 plays a role in the transport of transferrin receptors from early to recycling endosomes. ARL5B localizes to mitochondria and mediates retrograde trafficking from endosomes to the Golgi. ARF6 is mainly localized at the cell periphery and is involved in the recycling of endosomes. ARL8A and ARL8B best known function is the regulation of lysosome anterograde movement. ARL13B is a well-known regulator of cilia structure and function and promotes circular dorsal ruffle (CDR) formation and consequent cell migration. In BC, RAB2A, RAB5, RAB8, RAB40B and ARF6 participate in the transport of matrix metalloproteinases (MMPs) to invadopodia. ARF6 mediates epidermal growth factor receptor (EGFR) trafficking from the Golgi to the plasma membrane, promoting the progression of EGFR-dependent tumors. RAB40C, ARF1 and ARL13B control BC cell migration through the regulation of focal adhesions. ARF1 also regulates the formation of CDRs to promote cell migration. RAB27A controls the exocytosis of multivesicular bodies with consequent release of exosomes that contribute to BC progression. RAB21 and RAB34 are involved in the endocytosis and recycling of integrins to enhance cell migration. ARL8B mediates the transport of lysosomes to the cell periphery, which leads to their exocytosis with consequent release of proteases to the extracellular milieu. ARF4 regulates BC cell growth and invasion by mediating the transport of proteins from the Golgi to the ER. RAB26 reduces focal adhesion association of Src kinase and induces the degradation of phosphorylated Src, resulting in the inhibition of migration and invasion of BC cells. Created with BioRender.

BC remains a deadly disease and a challenge at the therapeutic level. We now know several intracellular pathways controlled by different RABs and ARFs that are subverted in BC and others could be uncovered in the future (Figure 2). Importantly, the modulation of RAB/ARF expression, activity and function holds the potential to provide novel therapeutic strategies and/or BC progression markers. However, several hurdles remain to be overcome, including the identification of effector molecules and further characterization of the mechanisms involved in the changes in membrane trafficking; the uncovering of the role played by the crosstalk between RABs, ARFs and other RAS superfamily members; and a better knowledge about the influence of membrane assemblies and dynamics in the alterations observed. While direct targeting of RABs and ARFs has proven challenging, alternative methods to inhibit these proteins are starting to emerge. These approaches include the impairment of nucleotide binding, hindering the interaction between small GTPases and their effectors, which blocks the recruitment to specific membrane sites, as well as the targeting of their regulators such as GEFs, GAPs or post-translational modification enzymes. Although more challenging, modulation of expression RABs/ARFs could also be used as a strategy to impair BC progression. Despite their limitations, these novel strategies have shown encouraging results and are gaining recognition. Consequently, further studies are essential to identify new and more specific inhibitors and assess their potential in preventing BC progression.

## References

[B1] Adarska P. Wong-Dilworth L. Bottanelli F. (2021). ARF GTPases and their ubiquitous role in intracellular trafficking beyond the Golgi. Front. Cell Dev. Biol. 9, 679046–679047. 10.3389/fcell.2021.679046 34368129 PMC8339471

[B153] Agola J. O. Hong L. Surviladze Z. Ursu O. Waller A. Strouse J. J. (2012). A competitive nucleotide binding inhibitor: in vitro characterization of Rab7 GTPase inhibition. ACS Chem. Biol. 7 (6), 1095–1108. 10.1021/cb3001099 22486388 PMC3440014

[B2] Allaire P. D. Sadr M. S. Chaineau M. Sadr E. S. Konefal S. Fotouhi M. (2013). Interplay between Rab35 and Arf6 controls cargo recycling to coordinate cell adhesion and migration. J. Cell Sci. 126, 722–731. 10.1242/jcs.112375 23264734

[B3] Alory C. Balch W. E. (2003). Molecular evolution of the rab-escort-protein/guanine-nucleotide-dissociation-inhibitor superfamily. Mol. Biol. Cell 14, 3857–3867. 10.1091/e03-04-0227 12972569 PMC196578

[B4] Arimoto K. I. Funami K. Saeki Y. Tanaka K. Okawa K. Takeuchi O. (2010). Polyubiquitin conjugation to NEMO by triparite motif protein 23 (TRIM23) is critical in antiviral defense. Proc. Natl. Acad. Sci. U. S. A. 107, 15856–15861. 10.1073/pnas.1004621107 20724660 PMC2936632

[B5] Arrazola Sastre A. Luque Montoro M. Lacerda H. M. Llavero F. Zugaza J. L. (2021). Small GTPases of the rab and arf families: key regulators of intracellular trafficking in neurodegeneration. Int. J. Mol. Sci. 22, 4425. 10.3390/ijms22094425 33922618 PMC8122874

[B6] Arya S. B. Kumar G. Kaur H. Kaur A. Tuli A. (2018). ARL11 regulates lipopolysaccharide-stimulated macrophage activation by promoting mitogen-activated protein kinase (MAPK) signaling. J. Biol. Chem. 293, 9892–9909. 10.1074/jbc.RA117.000727 29618517 PMC6016484

[B7] Avanzato D. Pupo E. Ducano N. Isella C. Bertalot G. Luise C. (2018). High USP6NL levels in breast cancer sustain chronic AKT phosphorylation and GLUT1 stability fueling aerobic glycolysis. Cancer Res. 78, 3432–3444. 10.1158/0008-5472.CAN-17-3018 29691252

[B8] Baron R. A. Seabra M. C. (2008). Rab geranylgeranylation occurs preferentially via the pre-formed REP-RGGT complex and is regulated by geranylgeranyl pyrophosphate. Biochem. J. 415, 67–75. 10.1042/BJ20080662 18532927

[B9] Beghin A. Belin S. Sleiman R. H. Manquat S. B. Goddard S. Tabone E. (2009). ADP ribosylation factor like 2 (Arl2) regulates breast tumor aggressivity in immunodeficient mice. PLoS One 4, e7478. 10.1371/journal.pone.0007478 19829707 PMC2759505

[B10] Beghin A. Honore S. Messana C. Matera E. Aim J. Burlinchon S. (2007). ADP ribosylation factor like 2 (Arl2) protein influences microtubule dynamics in breast cancer cells. Exp. Cell Res. 313, 473–485. 10.1016/j.yexcr.2006.10.024 17188265

[B11] Beghin A. Matera E.-L. Brunet-Manquat S. Dumontet C. (2008). Expression of Arl2 is associated with p53 localization and chemosensitivity in a breast cancer cell line. Cell Cycle 7, 3074–3082. 10.4161/cc.7.19.6777 18818514

[B12] Bhamidipati A. Lewis S. A. Cowan N. J. (2000). ADP ribosylation factor-like protein 2 (Arl2) regulates the interaction of tubulin-folding cofactor D with native tubulin. J. Cell Biol. 149, 1087–1096. 10.1083/jcb.149.5.1087 10831612 PMC2174823

[B13] Bobrie A. Krumeich S. Reyal F. Recchi C. Moita L. F. Seabra M. C. (2012). Rab27a supports exosome-dependent and -independent mechanisms that modify the tumor microenvironment and can promote tumor progression. Cancer Res. 72, 4920–4930. 10.1158/0008-5472.CAN-12-0925 22865453

[B14] Bravo-Cordero J. J. Marrero-Diaz R. Megías D. Genís L. García-Grande A. García M. A. (2007). MT1-MMP proinvasive activity is regulated by a novel Rab8-dependent exocytic pathway. EMBO J. 26, 1499–1510. 10.1038/sj.emboj.7601606 17332756 PMC1829373

[B15] Calin G. A. Trapasso F. Shimizu M. Dumitru C. D. Yendamuri S. Godwin A. K. (2005). Familial cancer associated with a polymorphism in ARLTS1. New Englang J. Med. 352, 1667–1676. 10.1056/NEJMoa042280 15843669

[B16] Casalou C. Faustino A. Barral D. C. (2016). Arf proteins in cancer cell migration. Small GTPases 7, 270–282. 10.1080/21541248.2016.1228792 27589148 PMC5129889

[B17] Casalou C. Faustino A. Silva F. Ferreira I. C. Vaqueirinho D. Ferreira A. (2019). Arl13b regulates breast cancer cell migration and invasion by controlling integrin-mediated signaling. Cancers (Basel) 11, 1461. 10.3390/cancers11101461 31569511 PMC6826833

[B18] Casalou C. Ferreira A. Barral D. C. (2020). The role of ARF family proteins and their regulators and effectors in cancer progression: a therapeutic perspective. Front. Cell Dev. Biol. 8, 217–313. 10.3389/fcell.2020.00217 32426352 PMC7212444

[B19] Casalou C. Seixas C. Portelinha A. Pintado P. Barros M. Ramalho J. S. (2014). Arl13b and the non-muscle myosin heavy chain IIA are required for circular dorsal ruffle formation and cell migration. J. Cell Sci. 127, 2709–2722. 10.1242/jcs.143446 24777479

[B20] Chang J. Yang R. Chen L. Fan Z. Zhou J. Guo H. (2022). Discovery of ARF1-targeting inhibitor demethylzeylasteral as a potential agent against breast cancer. Acta Pharm. Sin. B 12, 2619–2622. 10.1016/j.apsb.2022.02.011 35646528 PMC9136604

[B21] Chen P. I. Kong C. Su X. Stahl P. D. (2009). Rab5 isoforms differentially regulate the trafficking and degradation of epidermal growth factor receptors. J. Biol. Chem. 284, 30328–30338. 10.1074/jbc.M109.034546 19723633 PMC2781588

[B22] Chen P. W. Gasilina A. Yadav M. P. Randazzo P. A. (2022). Control of cell signaling by Arf GTPases and their regulators: focus on links to cancer and other GTPase families. Biochim. Biophys. Acta - Mol. Cell Res. 1869, 119171. 10.1016/j.bbamcr.2021.119171 34774605

[B23] Chen W. Feng Y. Chen D. Wandinger-Ness A. (1998). Rab11 is required for trans-Golgi network-to-plasma membrane transport and a preferential target for GDP dissociation inhibitor. Mol. Biol. Cell 9, 3241–3257. 10.1091/mbc.9.11.3241 9802909 PMC25617

[B24] Cheng J. M. Volk L. Janaki D. K. M. Vyakaranam S. Ran S. Rao K. A. (2010). Tumor suppressor function of Rab25 in triple-negative breast cancer. Int. J. Cancer 126, 2799–2812. 10.1002/ijc.24900 19795443

[B25] Deng W. Wang Y. Gu L. Duan B. Cui J. Zhang Y. (2016). MICAL1 controls cell invasive phenotype via regulating oxidative stress in breast cancer cells. BMC Cancer 16, 489–511. 10.1186/s12885-016-2553-1 27430308 PMC4950114

[B26] Derycke L. Stove C. Wever O. D. E. Dollé L. Colpaert N. Depypere H. (2011). The role of non-muscle myosin IIA in aggregation and invasion of human MCF-7 breast cancer cells. Int. J. Dev. Biol. 55, 835–840. 10.1387/ijdb.113336ld 22161839

[B27] Donaldson J. G. (2003). Multiple roles for Arf6: sorting, structuring, and signaling at the plasma membrane. J. Biol. Chem. 278, 41573–41576. 10.1074/jbc.R300026200 12912991

[B28] Donaldson J. G. Jackson C. L. (2011). ARF family G proteins and their regulators: roles in membrane transport, development and disease. Nat. Rev. Mol. Cell Biol. 12, 362–375. 10.1038/nrm3117 21587297 PMC3245550

[B29] Dong W. He B. Qian H. Liu Q. Wang D. Li J. (2018). RAB26-dependent autophagy protects adherens junctional integrity in acute lung injury. Autophagy 14, 1677–1692. 10.1080/15548627.2018.1476811 29965781 PMC6135632

[B30] Duan X. Xu L. Li Y. Jia L. Liu W. Shao W. (2021). Regulation of lipid homeostasis by the TBC protein dTBC1D22 via modulation of the small GTPase Rab40 to facilitate lipophagy. Cell Rep. 36, 109541. 10.1016/j.celrep.2021.109541 34469730

[B31] Encarnação M. Espada L. Escrevente C. Mateus D. Ramalho J. Michelet X. (2016). A Rab3a-dependent complex essential for lysosome positioning and plasma membrane repair. J. Cell Biol. 213, 631–640. 10.1083/jcb.201511093 27325790 PMC4915190

[B32] Escrevente C. Bento-Lopes L. Ramalho J. S. Barral D. C. (2021). Rab11 is required for lysosome exocytosis through the interaction with Rab3a, Sec15 and GRAB. J. Cell Sci. 134, jcs246694. 10.1242/jcs.246694 34100549 PMC8214760

[B33] Francis J. W. Newman L. E. Cunningham L. A. Kahn R. A. (2017). A trimer consisting of the tubulin-specific chaperone D (TBCD), regulatory GTPase ARL2, and β-tubulin is required for maintaining the microtubule network. J. Biol. Chem. 292, 4336–4349. 10.1074/jbc.M116.770909 28126905 PMC5354482

[B34] Frank B. Hemminki K. Meindl A. Wappenschmidt B. Klaes R. Schmutzler R. K. (2006). Association of the ARLTS1 Cys148Arg variant with familial breast cancer risk. Int. J. Cancer 118, 2505–2508. 10.1002/ijc.21687 16353159

[B35] Frittoli E. Palamidessi A. Marighetti P. Confalonieri S. Bianchi F. Malinverno C. (2014). A RAB5/RAB4 recycling circuitry induces a proteolytic invasive program and promotes tumor dissemination. J. Cell Biol. 206, 307–328. 10.1083/jcb.201403127 25049275 PMC4107781

[B36] Gao Z. Yang Y. Y. Huang M. Qi T. F. Wang H. Wang Y. (2022). Targeted proteomic analysis of small GTPases in radioresistant breast cancer cells. Anal. Chem. 94, 14925–14930. 10.1021/acs.analchem.2c02389 36264766 PMC9869664

[B37] Giubilaro J. Schuetz D. A. Stepniewski T. M. Namkung Y. Khoury E. Lara-Márquez M. (2021). Discovery of a dual Ras and ARF6 inhibitor from a GPCR endocytosis screen. Nat. Commun. 12, 4688. 10.1038/s41467-021-24968-y 34344896 PMC8333425

[B38] Goud B. Liu S. Storrie B. (2018). Rab proteins as major determinants of the Golgi complex structure. Small GTPases 9, 66–75. 10.1080/21541248.2017.1384087 29099310 PMC5902205

[B39] Guo H. Wang J. Ren S. Zheng L. F. Zhuang Y. X. Li D. L. (2022). Targeting EGFR-dependent tumors by disrupting an ARF6-mediated sorting system. Nat. Commun. 13, 6004–6015. 10.1038/s41467-022-33788-7 36224181 PMC9556547

[B40] Han K. J. Mikalayeva V. Gerber S. A. Kettenbach A. N. Skeberdis V. A. Prekeris R. (2022). Rab40c regulates focal adhesions and PP6 activity by controlling ANKRD28 ubiquitylation. Life Sci. Alliance 5, e202101346. 10.26508/lsa.202101346 35512830 PMC9070665

[B41] Hashimoto S. Onodera Y. Hashimoto A. Tanaka M. Hamaguchi M. Yamada A. (2004). Requirement for Arf6 in breast cancer invasive activities. Proc. Natl. Acad. Sci. 101, 6647–6652. 10.1073/pnas.0401753101 15087504 PMC404099

[B42] Hendrix A. Maynard D. Pauwels P. Braems G. Denys H. Van Den Broecke R. (2010). Effect of the secretory small GTPase Rab27B on breast cancer growth, invasion, and metastasis. J. Natl. Cancer Inst. 102, 866–880. 10.1093/jnci/djq153 20484105 PMC2886092

[B43] Hesse D. Hommel A. Jaschke A. Moser M. Bernhardt U. Zahn C. (2010). Altered GLUT4 trafficking in adipocytes in the absence of the GTPase Arfrp1. Biochem. Biophys. Res. Commun. 394, 896–903. 10.1016/j.bbrc.2010.03.059 20230794

[B44] Hong L. Guo Y. Basuray S. Agola J. O. Romero E. Simpson D. S. (2015). A Pan-GTPase inhibitor as a molecular probe. PLoS One 10, e0134317. 10.1371/journal.pone.0134317 26247207 PMC4527730

[B45] Howe E. N. Burnette M. D. Justice M. E. Schnepp P. M. Hedrick V. Clancy J. W. (2020). Rab11b-mediated integrin recycling promotes brain metastatic adaptation and outgrowth. Nat. Commun. 11, 3017–3115. 10.1038/s41467-020-16832-2 32541798 PMC7295786

[B46] Howley B. V. Link L. A. Grelet S. El-sabban M. Howe P. H. (2018). A CREB3-regulated ER-Golgi trafficking signature promotes metastatic progression in breast cancer. Oncogene 37, 1308–1325. 10.1038/s41388-017-0023-0 29249802 PMC5844805

[B47] Hu Z. Xu R. Liu J. Zhang Y. Du J. Li W. (2013). GEP100 regulates epidermal growth factor-induced MDA-MB-231 breast cancer cell invasion through the activation of Arf6/ERK/uPAR signaling pathway. Exp. Cell Res. 319, 1932–1941. 10.1016/j.yexcr.2013.05.028 23747719

[B48] Huang D. Pei Y. Dai C. Huang Y. Chen H. Chen X. (2019). Up-regulated ADP-Ribosylation factor 3 promotes breast cancer cell proliferation through the participation of FOXO1. Exp. Cell Res. 384, 111624. 10.1016/j.yexcr.2019.111624 31539530

[B49] Huang X. Lai S. Qu F. Li Z. Fu X. Li Q. (2022). CCL18 promotes breast cancer progression by exosomal miR-760 activation of ARF6/Src/PI3K/Akt pathway. Mol. Ther. - Oncolytics 25, 1–15. 10.1016/j.omto.2022.03.004 35399607 PMC8971730

[B50] Ioannou M. S. Bell E. S. Girard M. Chaineau M. Hamlin J. N. R. Daubaras M. (2015). DENND2B activates Rab13 at the leading edge of migrating cells and promotes metastatic behavior. J. Cell Biol. 208, 629–648. 10.1083/jcb.201407068 25713415 PMC4347646

[B51] Ioannou M. S. McPherson P. S. (2016). Regulation of cancer cell behavior by the small GTPase Rab13. J. Biol. Chem. 291, 9929–9937. 10.1074/jbc.R116.715193 27044746 PMC4858996

[B52] Ishida M. Bonifacino J. S. (2019). ARFRP1 functions upstream of ARL1 and ARL5 to coordinate recruitment of distinct tethering factors to the trans-Golgi network. J. Cell Biol. 218, 3681–3696. 10.1083/jcb.201905097 31575603 PMC6829661

[B53] Jacob A. Jing J. Lee J. Schedin P. Gilbert S. M. Peden A. A. (2013). Rab40b regulates trafficking of MMP2 and MMP9 during invadopodia formation and invasion of breast cancer cells. J. Cell Sci. 126, 4647–4658. 10.1242/jcs.126573 23902685 PMC3795337

[B54] Jacob A. Linklater E. Bayless B. A. Lyons T. Prekeris R. (2016). The role and regulation of Rab40b-Tks5 complex during invadopodia formation and cancer cell invasion. J. Cell Sci. 129, 4341–4353. 10.1242/jcs.193904 27789576 PMC5201011

[B55] Jang S. Y. Jang S. Ko J. (2012). Regulation of ADP-ribosylation factor 4 expression by small leucine zipper protein and involvement in breast cancer cell migration. Cancer Lett. 314, 185–197. 10.1016/j.canlet.2011.09.028 22004728

[B56] Jin H. Tang Y. Yang L. Peng X. Li B. Fan Q. (2021). Rab GTPases: central coordinators of membrane trafficking in cancer. Front. Cell Dev. Biol. 9, 648384–648413. 10.3389/fcell.2021.648384 34141705 PMC8204108

[B57] Kahn R. A. Cherfils J. Elias M. Lovering R. C. Munro S. Schurmann A. (2006). Nomenclature for the human Arf family of GTP-binding proteins: ARF, ARL, and SAR proteins. J. Cell Biol. 172, 645–650. 10.1083/jcb.200512057 16505163 PMC2063696

[B58] Kahn R. A. East M. P. Francis J. W. (2014). “ARF-like (ARL) proteins,” in Ras superfamily small G proteins: Biology and mechanisms 2. Editor Wittinghofer A. (Cham: Springer International Publishing), 215–251. 10.1007/978-3-319-07761-1_10

[B59] Kajiho H. Kajiho Y. Frittoli E. Confalonieri S. Bertalot G. Viale G. (2016). RAB2A controls MT1-MMP endocytic and E-cadherin polarized Golgi trafficking to promote invasive breast cancer programs. EMBO Rep. 17, 1061–1080. 10.15252/embr.201642032 27255086 PMC4931572

[B60] Kajiho H. Kajiho Y. Scita G. (2017). Harnessing membrane trafficking to promote cancer spreading and invasion: the case of RAB2A. Small GTPases 1248, 304–309. 10.1080/21541248.2016.1223990 PMC5997154 28060560

[B61] Kasmapour B. Gronow A. Bleck C. K. E. Hong W. Gutierrez M. G. (2012). Size-dependent mechanism of cargo sorting during lysosome-phagosome fusion is controlled by Rab34. Proc. Natl. Acad. Sci. U. S. A. 109, 20485–20490. 10.1073/pnas.1206811109 23197834 PMC3528610

[B62] Kelly E. E. Horgan C. P. McCaffrey M. W. (2012). Rab11 proteins in health and disease. Biochem. Soc. Trans. 40, 1360–1367. 10.1042/BST20120157 23176481

[B63] Keren-Kaplan T. Sarić A. Ghosh S. Williamson C. D. Jia R. Li Y. (2022). RUFY3 and RUFY4 are ARL8 effectors that promote coupling of endolysosomes to dynein-dynactin. Nat. Commun. 13, 1506. 10.1038/s41467-022-28952-y 35314674 PMC8938451

[B64] Khatter D. Sindhwani A. Sharma M. (2015). Arf-like GTPase Arl8: moving from the periphery to the center of lysosomal biology. Cell. Logist. 5, e1086501. 10.1080/21592799.2015.1086501 27057420 PMC4820812

[B65] Klinkert K. Echard A. (2016). Rab35 GTPase: a central regulator of phosphoinositides and F-actin in endocytic recycling and beyond. Traffic 17, 1063–1077. 10.1111/tra.12422 27329675

[B66] Kögel T. Rudolf R. Hodneland E. Copier J. Regazzi R. Tooze S. A. (2013). Rab3D is critical for secretory granule maturation in PC12 cells. PLoS One 8, e57321. 10.1371/journal.pone.0057321 23526941 PMC3602456

[B67] Kothari C. Clemenceau A. Ouellette G. Ennour-Idrissi K. Michaud A. C.-Gaudreault R. (2021). Tbc1d9: an important modulator of tumorigenesis in breast cancer. Cancers (Basel) 13, 3557. 10.3390/cancers13143557 34298771 PMC8304074

[B68] Kumar G. Chawla P. Dhiman N. Chadha S. Sharma S. Sethi K. (2022). RUFY3 links Arl8b and JIP4-Dynein complex to regulate lysosome size and positioning. Nat. Commun. 13, 1540. 10.1038/s41467-022-29077-y 35314681 PMC8938454

[B69] Leung K. F. Baron R. Seabra M. C. (2006). Thematic review series: lipid posttranslational modifications. geranylgeranylation of Rab GTPases. J. Lipid Res. 47, 467–475. 10.1194/jlr.R500017-JLR200 16401880

[B70] Li C. Fan Y. Lan T. H. Lambert N. A. Wu G. (2012). Rab26 modulates the cell surface transport of α2-adrenergic receptors from the Golgi. J. Biol. Chem. 287, 42784–42794. 10.1074/jbc.M112.410936 23105096 PMC3522277

[B71] Li L. Sun R. M. Jiang G. Q. (2020). ATF3 demethylation promotes the transcription of ARL4C, which acts as a tumor suppressor in human breast cancer. Onco. Targets. Ther. 13, 3467–3476. 10.2147/OTT.S243632 32425548 PMC7195577

[B72] Li R. Peng C. Zhang X. Wu Y. Pan S. Xiao Y. (2017a). Roles of Arf6 in cancer cell invasion, metastasis and proliferation. Life Sci. 182, 80–84. 10.1016/j.lfs.2017.06.008 28625359

[B73] Li T. Guo Y. (2022). ADP-ribosylation factor family of small GTP-binding proteins: their membrane recruitment, activation, crosstalk and functions. Front. Cell Dev. Biol. 10, 813353–813359. 10.3389/fcell.2022.813353 35186926 PMC8850633

[B74] Li W. Li G. Fan Z. Liu T. (2017b). Tumor-suppressive microRNA-452 inhibits migration and invasion of breast cancer cells by directly targeting RAB11A. Oncol. Lett. 14, 2559–2565. 10.3892/ol.2017.6426 28781694 PMC5530176

[B75] Li Y. Kelly W. G. Logsdon J. M. Schurko A. M. Harfe B. D. Hill-Harfe K. L. (2004). Functional genomic analysis of the ADP-ribosylation factor family of GTPases: phylogeny among diverse eukaryotes and function in *C. elegans* . FASEB J. 18, 1834–1850. 10.1096/fj.04-2273com 15576487

[B76] Li Z. Fang R. Fang J. He S. Liu T. (2018). Functional implications of Rab27 GTPases in cancer. Cell Commun. Signal. 16, 44–11. 10.1186/s12964-018-0255-9 30081925 PMC6080553

[B77] Liu H. Zhou Y. Qiu H. Zhuang R. Han Y. Liu X. (2021). Rab26 suppresses migration and invasion of breast cancer cells through mediating autophagic degradation of phosphorylated Src. Cell Death Dis. 12, 284. 10.1038/s41419-021-03561-7 33731709 PMC7969620

[B78] Liu Y. Zhang Z. Gao X. Ma Q. Yu Z. Huang S. (2022). Rab8A promotes breast cancer progression by increasing surface expression of Tropomyosin-related kinase B. Cancer Lett. 535, 215629. 10.1016/j.canlet.2022.215629 35278612

[B79] Loskutov Y. V. Kozyulina P. Y. Kozyreva V. K. Ice R. J. Jones B. C. Roston T. J. (2015). NEDD9/Arf6-dependent endocytic trafficking of matrix metalloproteinase 14: a novel mechanism for blocking mesenchymal cell invasion and metastasis of breast cancer. Oncogene 34, 3662–3675. 10.1038/onc.2014.297 25241893 PMC4369482

[B80] Luchsinger C. Aguilar M. Burgos P. V. Ehrenfeld P. Mardones G. A. (2018). Functional disruption of the Golgi apparatus protein ARF1 sensitizes MDA-MB-231 breast cancer cells to the antitumor drugs Actinomycin D and Vinblastine through ERK and AKT signaling. PLoS One 13, e0195401. 10.1371/journal.pone.0195401 29614107 PMC5882166

[B81] Machado E. White-Gilbertson S. Van De Vlekkert D. Janke L. Moshiach S. Campos Y. (2015). Regulated lysosomal exocytosis mediates cancer progression. Sci. Adv. 1, e1500603. 10.1126/sciadv.1500603 26824057 PMC4730843

[B82] Macia E. Vazquez-Rojas M. Robiolo A. Fayad R. Abélanet S. Mus-Veteau I. (2021). Chlortetracycline, a novel ARF inhibitor that decreases the ARF6-dependent invasive properties of breast cancer cells. Molecules 26, 969. 10.3390/molecules26040969 33673086 PMC7917842

[B83] Makki J. (2015). Diversity of breast carcinoma: histological subtypes and clinical relevance. Clin. Med. Insights Pathol. 8, 23–31. 10.4137/CPath.s31563 26740749 PMC4689326

[B84] Marchesin V. Castro-Castro A. Lodillinsky C. Castagnino A. Cyrta J. Bonsang-Kitzis H. (2015). ARF6-JIP3/4 regulate endosomal tubules for MT1-MMP exocytosis in cancer invasion. J. Cell Biol. 211, 339–358. 10.1083/jcb.201506002 26504170 PMC4621834

[B85] Marwaha R. Dwivedi D. Sharma M. (2019). Emerging roles of arf-like GTP-binding proteins: from membrane trafficking to cytoskeleton dynamics and beyond. Proc. Indian Natl. Sci. Acad. 85, 189–212. 10.16943/ptinsa/2019/49574

[B86] Matos P. (2021). Small GTPases in cancer: still signaling the way. Cancers (Basel) 13, 1500. 10.3390/cancers13071500 33805854 PMC8037031

[B87] Millar A. L. Pavlos N. J. Xu J. Zheng M. H. (2002). Rab3D: a regulator of exocytosis in non-neuronal cells. Histol. Histopathol. 17, 929–936. 10.14670/HH-17.929 12168804

[B88] Mima J. (2018). Reconstitution of membrane tethering mediated by Rab-family small GTPases. Biophys. Rev. 10, 543–549. 10.1007/s12551-017-0358-3 29204879 PMC5899718

[B89] Mitra S. Federico L. Zhao W. Dennison J. Sarkar T. R. Zhang F. (2016). Rab25 acts as an oncogene in luminal B breast cancer and is causally associated with Snail driven EMT. Oncotarget 7, 40252–40265. 10.18632/oncotarget.9730 27259233 PMC5130006

[B90] Mitra S. Montgomery J. E. Kolar M. J. Li G. Jeong K. J. Peng B. (2017). Stapled peptide inhibitors of RAB25 target context-specific phenotypes in cancer. Nat. Commun. 8, 660. 10.1038/s41467-017-00888-8 28939823 PMC5610242

[B91] Morishige M. Hashimoto S. Ogawa E. Toda Y. Kotani H. Hirose M. (2008). GEP100 links epidermal growth factor receptor signalling to Arf6 activation to induce breast cancer invasion. Nat. Cell Biol. 10, 85–92. 10.1038/ncb1672 18084281

[B92] Nashida T. Imai A. Shimomura H. (2006). Relation of Rab26 to the amylase release from rat parotid acinar cells. Arch. Oral Biol. 51, 89–95. 10.1016/j.archoralbio.2005.06.005 16076461

[B93] Nawrotek A. Benabdi S. Niyomchon S. Kryszke M. H. Ginestier C. Cañeque T. (2019). PH-domain-binding inhibitors of nucleotide exchange factor BRAG2 disrupt Arf GTPase signaling. Nat. Chem. Biol. 15, 358–366. 10.1038/s41589-019-0228-3 30742123

[B94] Newman L. E. Schiavon C. R. Zhou C. Kahn R. A. (2017). The abundance of the ARL2 GTPase and its GAP, ELMOD2, at mitochondria are modulated by the fusogenic activity of mitofusins and stressors. PLoS One 12, e0175164. 10.1371/journal.pone.0175164 28380071 PMC5381910

[B95] Newman L. E. Zhou C. J. Mudigonda S. Mattheyses A. L. Paradies E. Marobbio C. M. T. (2014). The ARL2 GTPase is required for mitochondrial morphology, motility, and maintenance of ATP levels. PLoS One 9, 992700. 10.1371/journal.pone.0099270 PMC4050054 24911211

[B96] Ohashi Y. Iijima H. Yamaotsu N. Yamazaki K. Sato S. Okamura M. (2012). AMF-26, a novel inhibitor of the Golgi system, targeting ADP-ribosylation factor 1 (Arf1) with potential for cancer therapy. J. Biol. Chem. 287, 3885–3897. 10.1074/jbc.M111.316125 22158626 PMC3281721

[B97] Okamoto S. Jiang Y. Kawamura K. Shingyoji M. Tada Y. Sekine I. (2014). Zoledronic acid induces apoptosis and S-phase arrest in mesothelioma through inhibiting Rab family proteins and topoisomerase II actions. Cell Death Dis. 5, e1517. 10.1038/cddis.2014.475 25393473 PMC4260733

[B98] Palmieri D. Bouadis A. Ronchetti R. Merino M. J. Steeg P. S. (2006). Rab11a differentially modulates epidermal growth factor-induced proliferation and motility in immortal breast cells. Breast Cancer Res. Treat. 100, 127–137. 10.1007/s10549-006-9244-6 16791477

[B99] Park J. Matralis A. N. Berghuis A. M. Tsantrizos Y. S. (2014). Human isoprenoid synthase enzymes as therapeutic targets. Front. Chem. 2, 50–21. 10.3389/fchem.2014.00050 25101260 PMC4106277

[B100] Pellinen T. Arjonen A. Vuoriluoto K. Kallio K. Fransen J. A. M. Ivaska J. (2006). Small GTPase Rab21 regulates cell adhesion and controls endosomal traffic of beta1-integrins. J. Cell Biol. 173, 767–780. 10.1083/jcb.200509019 16754960 PMC2063892

[B101] Pennauer M. Buczak K. Prescianotto-Baschong C. Spiess M. (2021). Shared and specific functions of Arfs 1–5 at the Golgi revealed by systematic knockouts. J. Cell Biol. 221, e202106100. 10.1083/jcb.202106100 34749397 PMC8579194

[B102] Pereira-Leal J. B. Seabra M. C. (2000). The mammalian Rab family of small GTPases: definition of family and subfamily sequence motifs suggests a mechanism for functional specificity in the Ras superfamily. J. Mol. Biol. 301, 1077–1087. 10.1006/jmbi.2000.4010 10966806

[B103] Petrocca F. Iliopoulos D. Qin H. R. Nicoloso M. S. Yendamuri S. Wojcik S. E. (2006). Alterations of the tumor suppressor gene ARLTS1 in ovarian cancer. Cancer Res. 66, 10287–10291. 10.1158/0008-5472.CAN-06-2289 17079447

[B104] Pylypenko O. Hammich H. Yu I. M. Houdusse A. (2018). Rab GTPases and their interacting protein partners: structural insights into Rab functional diversity. Small GTPases 9, 22–48. 10.1080/21541248.2017.1336191 28632484 PMC5902227

[B105] Qin X. Zhang Y. He Y. Chen K. Zhang Y. Li P. (2021). Shear stress triggered circular dorsal ruffles formation to facilitate cancer cell migration. Arch. Biochem. Biophys. 709, 108967. 10.1016/j.abb.2021.108967 34157295

[B106] Raffaniello R. D. (2021). Rab3 proteins and cancer: exit strategies. J. Cell. Biochem. 122, 1295–1301. 10.1002/jcb.29948 33982832

[B107] Reiner D. J. Lundquist E. A. (2018). Small GTPases. WormBook, 1–65. 10.1895/wormbook.1.67.2 PMC6369420 27218782

[B108] Ren M. Xu G. Zeng J. De Lemos-Chiarandini C. Adesnik M. Sabatini D. D. (1998). Hydrolysis of GTP on rab11 is required for the direct delivery of transferrin from the pericentriolar recycling compartment to the cell surface but not from sorting endosomes. Proc. Natl. Acad. Sci. U. S. A. 95, 6187–6192. 10.1073/pnas.95.11.6187 9600939 PMC27621

[B109] Revenkova E. Liu Q. Luca Gusella G. Iomini C. (2018). The Joubert syndrome protein ARL13B binds tubulin to maintain uniform distribution of proteins along the ciliary membrane. J. Cell Sci. 131, jcs212324. 10.1242/jcs.212324 29592971 PMC5992585

[B110] Riedel D. Antonin W. Fernandez-Chacon R. Alvarez de Toledo G. Jo T. Geppert M. (2002). Rab3D is not required for exocrine exocytosis but for maintenance of normally sized secretory granules. Mol. Cell. Biol. 22, 6487–6497. 10.1128/mcb.22.18.6487-6497.2002 12192047 PMC135623

[B111] Roelofs A. J. Hulley P. A. Meijer A. Ebetino F. H. Russell R. G. G. Shipman C. M. (2006). Selective inhibition of Rab prenylation by a phosphonocarboxylate analogue of risedronate induces apoptosis, but not S-phase arrest, in human myeloma cells. Int. J. Cancer 119, 1254–1261. 10.1002/ijc.21977 16619218

[B112] Rosa-Ferreira C. Munro S. (2011). Arl8 and SKIP act together to link lysosomes to kinesin-1. Dev. Cell 21, 1171–1178. 10.1016/j.devcel.2011.10.007 22172677 PMC3240744

[B113] Sahgal P. Alanko J. Icha J. Paatero I. Hamidi H. Arjonen A. (2019). GGA2 and RAB13 promote activity-dependent β1-integrin recycling. J. Cell Sci. 132, jcs233387. 10.1242/jcs.233387 31076515

[B114] Saito K. Maeda M. Katada T. (2017). Regulation of the Sar1 GTPase cycle is necessary for large cargo secretion from the endoplasmic reticulum. Front. Cell Dev. Biol. 5, 75–78. 10.3389/fcell.2017.00075 28879181 PMC5572378

[B115] Sato T. Mushiake S. Kato Y. Sato K. Sato M. Takeda N. (2007). The Rab8 GTPase regulates apical protein localization in intestinal cells. Nature 448, 366–369. 10.1038/nature05929 17597763

[B116] Schlienger S. Alain R. Ramirez M. Claing A. (2015). ARF1 regulates adhesion of MDA-MB-231 invasive breast cancer cells through formation of focal adhesions. Cell. Signal. 27, 403–415. 10.1016/j.cellsig.2014.11.032 25530216

[B117] Schlienger S. Campbell S. Pasquin S. Gaboury L. Claing A. (2016). ADP-ribosylation factor 1 expression regulates epithelial-mesenchymal transition and predicts poor clinical outcome in triple-negative breast cancer. Oncotarget 7, 15811–15827. 10.18632/oncotarget.7515 26908458 PMC4941279

[B118] Schlüter O. M. Basu J. Südhof T. C. Rosenmund C. (2006). Rab3 superprimes synaptic vesicles for release: implications for short-term synaptic plasticity. J. Neurosci. 26, 1239–1246. 10.1523/JNEUROSCI.3553-05.2006 16436611 PMC6674574

[B119] Schlüter O. M. Khvotchev M. Jahn R. Südhof T. C. (2002). Localization versus function of Rab3 proteins: evidence for a common regulatory role in controlling fusion. J. Biol. Chem. 277, 40919–40929. 10.1074/jbc.M203704200 12167638

[B120] Seixas C. Choi S. Y. Polgar N. Umberger N. L. East M. P. Zuo X. (2016). Arl13b and the exocyst interact synergistically in ciliogenesis. Mol. Biol. Cell 27, 308–320. 10.1091/mbc.E15-02-0061 26582389 PMC4713133

[B121] Shaughnessy R. Echard A. (2018). Rab35 GTPase and cancer: linking membrane trafficking to tumorigenesis. Traffic 19, 247–252. 10.1111/tra.12546 29314576

[B122] Siegel R. L. Miller K. D. Wagle N. S. Jemal A. (2023). Cancer statistics, 2023. Ca. Cancer J. Clin. 73, 17–48. 10.3322/caac.21763 36633525

[B123] Simpson J. C. Griffiths G. Wessling-Resnick M. Fransen J. A. M. Bennett H. Jones A. T. (2004). A role for the small GTPase Rab21 in the early endocytic pathway. J. Cell Sci. 117, 6297–6311. 10.1242/jcs.01560 15561770

[B124] Spiegel J. Cromm P. M. Itzen A. Goody R. S. Grossmann T. N. Waldmann H. (2014). Direct targeting of rab-GTPase-effector interactions. Angew. Chem. - Int. Ed. 53, 2498–2503. 10.1002/anie.201308568 24481744

[B125] Starling G. P. Yip Y. Y. Sanger A. Morton P. E. Eden E. R. Dodding M. P. (2016). Folliculin directs the formation of a Rab34– RILP complex to control the nutrient‐dependent dynamic distribution of lysosomes. EMBO Rep. 17, 823–841. 10.15252/embr.201541382 27113757 PMC4893818

[B126] Stenmark H. (2009). Rab GTPases as coordinators of vesicle traffic. Nat. Rev. Mol. Cell Biol. 10, 513–525. 10.1038/nrm2728 19603039

[B127] Sun L. Xu X. Chen Y. Zhou Y. Tan R. Qiu H. (2018). Rab34 regulates adhesion, migration, and invasion of breast cancer cells. Oncogene 37, 3698–3714. 10.1038/s41388-018-0202-7 29622794

[B128] Sztul E. Chen P. Casanova J. E. Cherfils J. Dacks J. B. Lambright D. G. (2019). ARF GTPases and their GEFs and GAPs: concepts and challenges. Mol. Biol. Cell 30, 1249–1271. 10.1091/mbc.E18-12-0820 31084567 PMC6724607

[B129] Tanaka N. Sakamoto T. (2023). MT1-MMP as a key regulator of metastasis. Cells 12, 2187–2216. 10.3390/cells12172187 37681919 PMC10486781

[B130] Tian X. Jin R. U. Bredemeyer A. J. Oates E. J. Błażewska K. M. McKenna C. E. (2010). RAB26 and RAB3D are direct transcriptional targets of MIST1 that regulate exocrine granule maturation. Mol. Cell. Biol. 30, 1269–1284. 10.1128/mcb.01328-09 20038531 PMC2820885

[B131] Ullrich O. Reinsch S. Urbé S. Zerial M. Parton R. G. (1996). Rab11 regulates recycling through the pericentriolar recycling endosome. J. Cell Biol. 135, 913–924. 10.1083/jcb.135.4.913 8922376 PMC2133374

[B132] Wang B. Yang Z. Wang H. Cao Z. Zhao Y. Gong C. (2015). MicroRNA-320a inhibits proliferation and invasion of breast cancer cells by targeting RAB11A. Am. J. Cancer Res. 5, 2719–2729. 26609479 PMC4633901

[B133] Wang J. S. Wang F. B. Zhang Q. G. Shen Z. Z. Shao Z. M. (2008). Enhanced expression of Rab27A gene by breast cancer cells promoting invasiveness and the metastasis potential by secretion of insulin-like growth factor-II. Mol. Cancer Res. 6, 372–382. 10.1158/1541-7786.MCR-07-0162 18337447

[B134] Wang T. Hong W. (2005). Assay and functional properties of Rab34 interaction with rilp in lysosome morphogenesis. Methods Enzymol. 403, 675–687. 10.1016/S0076-6879(05)03058-2 16473629

[B135] Watanabe M. Takahashi H. Saeki Y. Ozaki T. Itoh S. Suzuki M. (2015). The E3 ubiquitin ligase TRIM23 regulates adipocyte differentiation via stabilization of the adipogenic activator PPARγ. Elife 4, e05615. 10.7554/eLife.05615 25905670 PMC4426667

[B136] Wei S. Xie C. Abe Y. Cai J. (2009). ADP-ribosylation factor like 7 (ARL7) interacts with alpha-tubulin and modulates intracellular vesicular transport. Biochem. Biophys. Res. Commun. 384, 352–356. 10.1016/j.bbrc.2009.04.125 19409876

[B137] Welz T. Wellbourne-Wood J. Kerkhoff E. (2014). Orchestration of cell surface proteins by Rab11. Trends Cell Biol. 24, 407–415. 10.1016/j.tcb.2014.02.004 24675420

[B138] Wennerberg K. Rossman K. L. Der C. J. (2005). The Ras superfamily at a glance. J. Cell Sci. 118, 843–846. 10.1242/jcs.01660 15731001

[B139] Wu P. H. Onodera Y. Giaccia A. J. Le Q. T. Shimizu S. Shirato H. (2020). Lysosomal trafficking mediated by Arl8b and BORC promotes invasion of cancer cells that survive radiation. Commun. Biol. 3, 620–715. 10.1038/s42003-020-01339-9 33110168 PMC7591908

[B140] Wu T. Sun L. Wang C. Yu P. Cheng L. Chen Y. (2021). Sevoflurane suppresses the migration, invasion, and epithelial-mesenchymal transition of breast cancer cells through the miR-139-5p/ARF6 Axis. J. Surg. Res. 258, 314–323. 10.1016/j.jss.2020.08.051 33317757

[B141] Xie X. Tang S. C. Cai Y. Pi W. Deng L. Wu G. (2016). Suppression of breast cancer metastasis through the inactivation of ADP-ribosylation factor 1. Oncotarget 7, 58111–58120. 10.18632/oncotarget.11185 27517156 PMC5295416

[B142] Yang J. Liu W. Lu X. Fu Y. Li L. Luo Y. (2015). High expression of small GTPase Rab3D promotes cancer progression and metastasis. Oncotarget 6, 11125–11138. 10.18632/oncotarget.3575 25823663 PMC4484444

[B143] Yendamuri S. Trapasso F. Calin G. A. (2008). ARLTS1 - a novel tumor suppressor gene. Cancer Lett. 264, 11–20. 10.1016/j.canlet.2008.02.021 18375053

[B144] Yin Y. X. Shen F. Pei H. Ding Y. Zhao H. Zhao M. (2012). Increased expression of Rab25 in breast cancer correlates with lymphatic metastasis. Tumor Biol. 33, 1581–1587. 10.1007/s13277-012-0412-5 22644676

[B145] Yoon S. O. Shin S. Mercurio A. M. (2005). Hypoxia stimulates carcinoma invasion by stabilizing microtubules and promoting the Rab11 trafficking of the alpha6beta4 integrin. Cancer Res. 65, 2761–2769. 10.1158/0008-5472.CAN-04-4122 15805276

[B146] Yuan W. Song C. (2020). The emerging role of Rab5 in membrane receptor trafficking and signaling pathways. Biochem. Res. Int. 2020, 4186308. 10.1155/2020/4186308 32104603 PMC7036122

[B147] Zhang J. Zhou Y. Yu Z.-H. Chen A. Yu Y. Wang X. (2019). Identification of core genes and clinical roles in pregnancy-associated breast cancer based on integrated analysis of different microarray profile datasets. Biosci. Rep. 25 (6), BSR20190019. 10.1042/BSR20190019 PMC6591572 31171715

[B148] Zhang J. X. Huang X. X. Cai M. B. Tong Z. T. Chen J. W. Qian D. (2012). Overexpression of the secretory small GTPase Rab27B in human breast cancer correlates closely with lymph node metastasis and predicts poor prognosis. J. Transl. Med. 10, 242–310. 10.1186/1479-5876-10-242 23217148 PMC3539959

[B149] Zhang K. Liu H. Song Z. Jiang Y. Kim H. Samavati L. (2020). The UPR transducer IRE1 promotes breast cancer malignancy by degrading tumor suppressor microRNAs. iScience 23, 101503. 10.1016/j.isci.2020.101503 32911332 PMC7490531

[B150] Zhao H. Ahirwar D. K. Oghumu S. Wilkie T. Powell C. A. Nasser M. W. (2016). Endothelial Robo4 suppresses breast cancer growth and metastasis through regulation of tumor angiogenesis. Mol. Oncol. 10, 272–281. 10.1016/j.molonc.2015.10.007 26778715 PMC4739522

[B151] Zhao Y. G. Zhang H. (2020). Phase separation in membrane biology: the interplay between membrane-bound organelles and membraneless condensates. Dev. Cell 55, 30–44. 10.1016/j.devcel.2020.06.033 32726575

[B152] Zhu Y. Shen T. Liu J. Zheng J. Zhang Y. Xu R. (2013). Rab35 is required for Wnt5a/Dvl2-induced Rac1 activation and cell migration in MCF-7 breast cancer cells. Cell. Signal 25, 1075–1085. 10.1016/j.cellsig.2013.01.015 23353182

